# Tuned near infrared fluorescent hyaluronic acid conjugates for delivery to pancreatic cancer for intraoperative imaging

**DOI:** 10.7150/thno.40688

**Published:** 2020-02-10

**Authors:** Bowen Qi, Ayrianne J. Crawford, Nicholas E. Wojtynek, Geoffrey A. Talmon, Michael A. Hollingsworth, Quan P. Ly, Aaron M. Mohs

**Affiliations:** 1 Department of Pharmaceutical Sciences, University of Nebraska Medical Center, Omaha, Nebraska 68198, United States; 2 Eppley Institute for Research in Cancer and Allied Diseases, University of Nebraska Medical Center, Omaha, Nebraska 68198, United States; 3 Department of Pathology and Microbiology, University of Nebraska Medical Center, Omaha, Nebraska 68198, United States; 4 Fred and Pamela Buffett Cancer Center, University of Nebraska Medical Center, Omaha, Nebraska 68198, United States; 5 Department of Surgery, University of Nebraska Medical Center, Omaha, Nebraska 68198, United States; 6 Department of Biochemistry and Molecular Biology, University of Nebraska Medical Center, Omaha, Nebraska 68198, United States

**Keywords:** Pancreatic ductal adenocarcinoma, hyaluronic acid, fluorescence guided surgery, biodistribution, serum protein binding

## Abstract

The prognosis of pancreatic cancer remains poor. Intraoperative fluorescence imaging of tumors could improve staging and surgical resection, thereby improving prognosis. However, imaging pancreatic cancer with macromolecular delivery systems, is often hampered by nonspecific organ accumulation.

**Methods**: We describe the rational development of hyaluronic acid (HA) conjugates that vary in molecular weight and are conjugated to near infrared fluorescent (NIRF) dyes that have differences in hydrophilicity, serum protein binding affinity, and clearance mechanism. We systematically investigated the roles of each of these properties on tumor accumulation, relative biodistribution, and the impact of intraoperative imaging of orthotopic, syngeneic pancreatic cancer.

**Results**: Each HA-NIRF conjugate displayed intrapancreatic tumor enhancement. Regardless of HA molecular weight, Cy7.5 conjugation directed biodistribution to the liver, spleen, and bowels. Conjugation of IRDye800 to 5 and 20 kDa HA resulted in low liver and spleen signal while enhancing the tumor up to 14-fold compared to healthy pancreas, while 100 kDa HA conjugated to IRDye800 resulting in liver and spleen accumulation.

**Conclusion**: These studies demonstrate that by tuning HA molecular weight and the physicochemical properties of the conjugated moiety, in this case a NIRF probe, peritoneal biodistribution can be substantially altered to achieve optimized delivery to tumors intraoperative abdominal imaging.

## Introduction

Surgical resection remains a promising treatment for improving prognosis of cancer patients as it is potentially curative for primary tumors and local metastases 1. Currently, surgery is performed without contrast-enhanced image-guidance to identify tumor margins and small occult metastases due to the absence of clinically available optical contrast agents 2. Contrast-enhanced optical imaging could provide real-time guidance during tumor resection with sensitive, rapid, and non-radioactive near-infrared fluorescent (NIRF) tracers to highlight the areas of extension of the malignancy 3-5. NIRF is promising for *in vivo* optical imaging due the relative biological transparency at wavelengths between 700 and 1000 nm 2. Fluorescence image-guided surgery (FIGS) has so far shown its potential to support surgical procedures and the feasibility to improve clinical outcomes 6-9. However, FIGS of tumors has not reached its full potential, in part due to contrast agents lacking high intensity, water solubility, biocompatibility, and tissue-specific targetability 10.

Attaining high contrast-to-noise ratio (CNR: signal in region of interest relative to neighboring region) and signal-to-noise ratio (SNR: signal in the region of interest relative to background noise) is vitally important for specific visual guidance of tumor detection and removal. Sufficient CNR and SNR are achieved due to fluorophore accumulation in target tissue, while unbound fluorophore is rapidly moved from healthy tissues and clearance organs 11. Nevertheless, most dyes are not tumor-specific and *in vivo* performance can be complicated by non-specific biological interaction, *e.g.* membranes, pharmacokinetic processes, including absorption, distribution, metabolism and excretion, and optical properties of the dye and tissue 12. For instance, some cyanine-based NIRF probes with hydrophobic core structures and high surface charge bind to plasma proteins, which results in reticuloendothelial (RES) organ sequestration and hepatobiliary clearance *in vivo*, which can compromise tumor contrast 13-16. Useful strategies for improving CNR and SNR include, among others, utilizing tumor or tumor microenvironment specific biomarkers 12 and altering excretion from the liver (hepatobiliary) to the kidneys (urinary) 17-19. Pancreatic cancer CNR was found to be significantly increased by targeting somatostatin 20,21 or tumor cell antigens 22 or utilizing cell-penetrating peptides 23 for tumor-specific enhancement. Reported by Choi, *et al.*^15^,^16^,^24^, improved tumor SNR was achieved by using zwitterionic fluorophores, which increased renal filtration and reduced overall background. Hence, we hypothesized that optimized pancreatic tumor contrast could be achieved by combining tumor-specific targeting and facilitating minimized intraperitoneal organ accumulation.

Previously, we reported the development of tumor-selective delivery of the FDA-approved NIR fluorophore, indocyanine green (ICG), by physically entrapping the dye in hyaluronic-acid (HA)-derived nanoformulations, termed NanoICG, in models of breast, prostate, and pancreatic cancer 25-28. NanoICG resulted in significant pancreatic cancer contrast relative to muscle reference and/or uninvolved pancreas, but also resulted in strong signal in intraperitoneal organs, especially the liver and spleen 28. To maximize pancreatic ductal adenocarcinoma (PDAC) contrast enhancement and to minimize RES capture, we sought to specifically examine the role of HA molecular weight and NIRF dye properties. Compared to our previous study of using hydrophobically-modified HA to drive nanoparticle self-assembly and physicochemical entrapment of ICG 28, two dyes, Cy7.5 and IRDye800, were directly conjugated to HA of three different molecular weights (MW_N_), including 5, 20, and 100 kDa. The effects of HA MW_N_ and physiological properties of NIR dyes on tumor specificity and biodistribution were investigated to minimize RES uptake, reduce peritoneal organ background fluorescence, and determine a lead agent for PDAC enhancement with minimized RES uptake. These select agents would ideally be useful for detection of intraperitoneal metastatic pancreatic cancer or other tumor types that require surgical detection or intervention in the abdominal cavity.

## Results and Discussion

### Photochemical characterization of HA-dye conjugates

**Figure 1:**
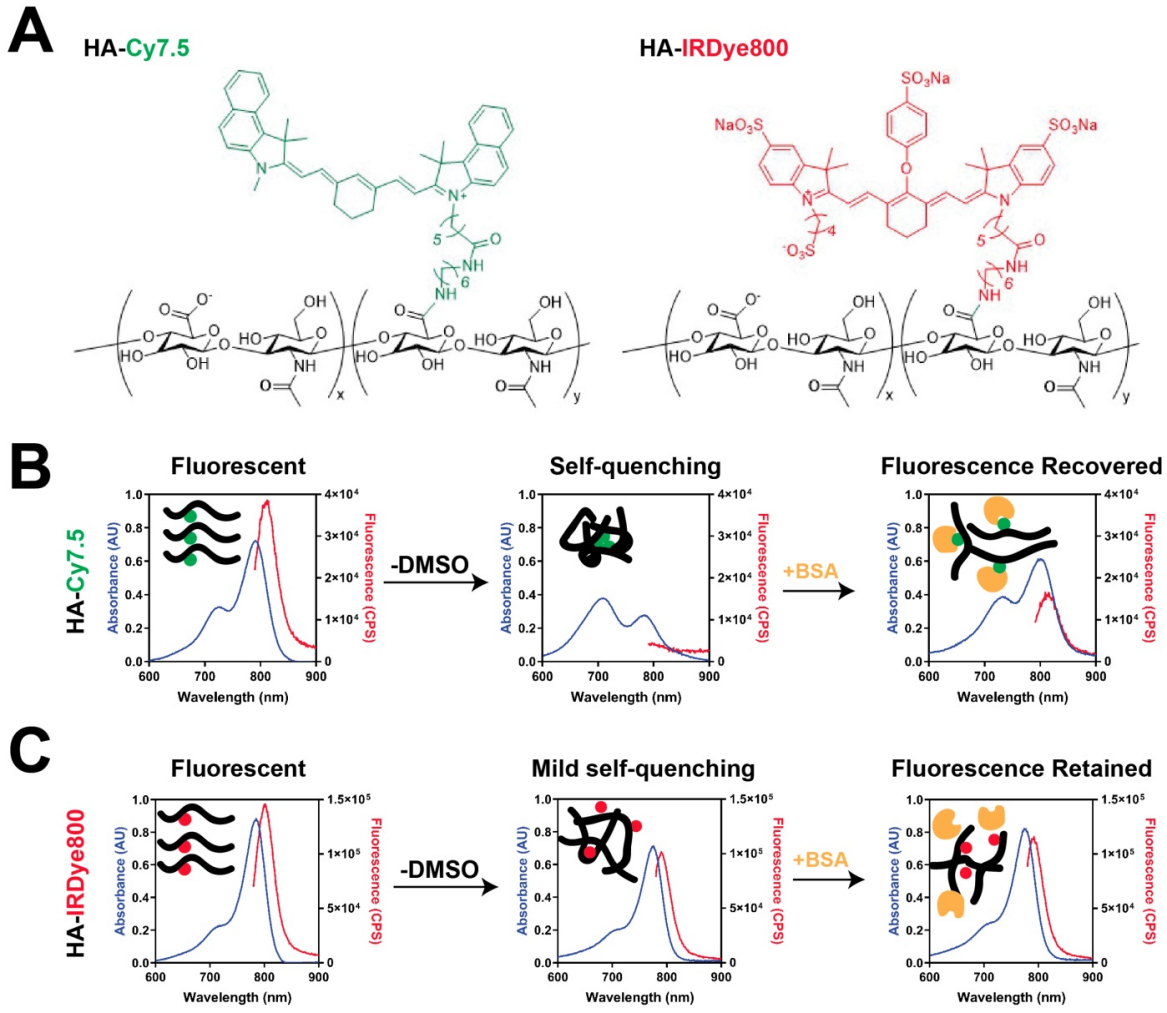
Characterization of HA-dyes with different HA MW_N_. (**A**) Chemical structures of HA-Cy7.5 and HA-IRDye800; x and y indicate the calculated number of disaccharide units and conjugated dyes per polymer chain, respectively; x = 9, y = 1 for HA_5k_-dye; x = 38, y = 1 for HA_20k_-dye; x = 169, y = 3 for HA_100k_-dye; (**B-C**) absorption and fluorescence emission pattern of HA-Cy7.5 and HA-IRDye800 in H_2_O, DMSO/H_2_O or BSA with corresponding schematic diagram of molecular interaction shown as insets; the absorption was collected between 600 nm to 900 nm, the fluorescence was excited at 775 nm for HA-Cy7.5 and 770 nm for HA-IRDye800, and collected between 790 nm to 900 nm for HA-Cy7.5 and 780 nm to 900 nm for HA-IRDye800. The concentration of free dye for HA-dye conjugates was consistent (4 μM) among solutions with and without DMSO and in BSA.

HA-Cy7.5 and HA-IRDye800 conjugates were synthesized with 1-Ethyl-3-(3-dimethylaminopropyl)-carbodiimide (EDC)/N-hydroxysuccinimide (NHS) coupling chemistry. The physicochemical and optical properties of HA-Cy7.5 and HA-IRDye800 are summarized in **Figure 1, Table S1,** and **Figure S1**. HA-Cy7.5 conjugates displayed distinct, solvent-dependent spectral properties: In H_2_O, fluorescent peaks were quenched with broad absorbance spectra compared to dissociation in DMSO, indicating self-assembly and self-quenching, which is consistent with nanoformulation of Cy7.5 29. Cyanine dyes are capable of forming H-aggregates by hydrophobic interaction, which is characterized with hypsochromic absorption and weak fluorescence emission 30-32. As shown in **Figure 1B** and **S1**, the absorption wavelength blue-shifted 80 nm for HA_5k_, HA_20k_ conjugated Cy7.5, while a blue shift of only 10 nm was observed for HA_100k_-Cy7.5 conjugate in H_2_O. Furthermore, upon disassembly in DMSO, the fluorescence emission intensity exhibited a red-shifted to 810 nm, and a 21.3-, 9.2- and 4.0- fold increase in fluorescence intensity for HA_5k_-, HA_20k_- and HA_100k_-dye conjugates, respectively. In comparison, hydrophilic IRDye800 displayed a limited tendency to interact with other IRDye800 molecules as opposed to interacting with polar solvent (**Figure 1C, Figure S1**). The negative charge also prevents close interaction of the heterocyclic rings between dyes, compromising π-π interaction. Therefore, conjugation of HA to IRDye800 had minimal impact on spectral properties of IRDye800 (**Figure 1C**).

**Figure 2:**
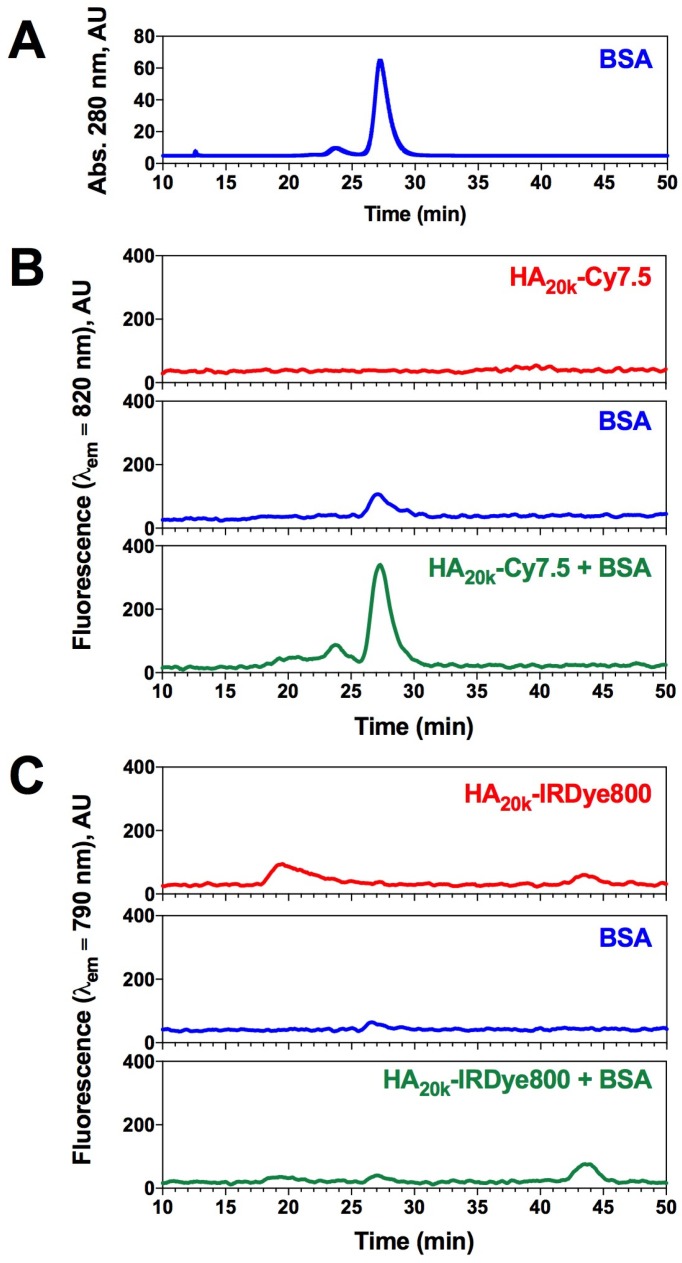
BSA has dye-selective interaction with HA-dye conjugates. (A) Elution chromatogram trace of 50 μM BSA monitored at 280 nm absorption; (B) SEC chromatogram of HA_20k_-Cy7.5 (4 uM Cy7.5 equivalent, red), BSA (50 uM, blue) or the mixture (green); λ_ex_ = 775 nm, λ_em_ = 820 nm, integration (int) = 5 s; (C) SEC chromatogram of HA_20k_-IRDye800 (4 uM IRDye800 equivalent, red), BSA (50 uM, blue) or the mixture (green); λ_ex_ = 770 nm, λ_em_ = 790 nm, int = 5 s.

It is worth noting that HA_20k_-dye conjugates are not nanoparticles (NPs) in aqueous phase. We examined the morphology and hydrodynamic diameter of HA_20k_-Cy7.5 with NP tracking analysis, dynamic light scattering, transmission electron microscopy and molecular docking, and concluded HA_20k_-Cy7.5 forms aggregates in the aqueous phase instead of self-assembled NPs (data not shown). BSA monomer is spherically shaped with a diameter of ~10 nm (**Figure S2A**). When mixed with HA_20k_-Cy7.5, BSA morphologically changed to irregular shape with sizes range from 10-70 nm (**Figure S2B**) and had elution time on size exclusion chromatography consistent with BSA (see **Figure 2**). In comparison, BSA monomer remains ~10 nm in the presence of HA_20k_-IRDye800. In summary, when conjugated with hydrophobic Cy7.5, HA-dye tends to aggregate and self-quench, but the fluorescence is recovered upon disassembly, or partially upon BSA interaction. No fluorescence quenching was observed with HA conjugated to the hydrophilic IRDye800, i.e., the spectral properties of HA-IRDye800 conjugates were conserved with or without BSA.

### Association of BSA with HA-dye conjugates

Plasma protein association can have a significant impact on *in vivo* size and solubility, which can alter pharmacokinetic properties and tissue specificity of systemically-administered agents 33. To further investigate whether conjugated dye caused non-specific protein association, a mixture of HA-dye and BSA were separated for protein recognition while being monitored for NIRF to identify the presence of HA-dye. ICG+BSA was used as a control of known NIRF-protein interaction to calibrate the chromatography-spectrophotometric system. The delay volume between UV detector and spectrofluorometer was 5.55 ± 0.18 mL (determined by FITC labeled dextran2000 and ICG+BSA peaks) and normalized in **Figure 2**.

BSA monomer was eluted maximally at 27 min when monitoring absorbance at 280 nm (**Figure 2A**), whereas minimal signal was observed in NIRF channel at that time (**Figure 2B-C,** blue trace). The NIRF of HA_20k_-Cy7.5 was undetectable due to self-quenching (**Figure 2B**, red trace), whereas HA_20k_-IRDye800 exhibits 2 peaks which likely correspond to aggregate and dispersed polymer conjugate (**Figure 2C**, red trace). Interestingly, a NIRF peak was detected when HA_20k_-Cy7.5 was mixed with BSA at the elution time of BSA (**Figure 2B,** green trace), which was consistent with the fluorescence recovery displayed spectrofluorometrically (**Figure 1B**). A corresponding peak from HA-IRDye800 attributed to BSA binding was minimal (**Figure 2C,** green trace). In order to confirm the presence of BSA, fractionated eluate of interest from HA-dye + BSA were collected for Bradford assay, the NIRF peak fractions were strongly positive after Bradford dye staining, indicating that NIRF peak of HA-Cy7.5 corresponds to BSA elution (data not shown).

Berezin, *et al.* 34 demonstrated that the fluorophore, but not the targeting moiety, is predominantly responsible for albumin binding of imaging probes. Meanwhile, HA is capable of reducing protein adsorption and potentially the immunogenicity of the protein corona 35,36. Cyanine dyes such as ICG, are able to bind with plasma proteins with high affinity, which results in complete extraction by hepatic parachyma and fast elimination into the bile 37,38**.** More importantly, after examining several NIR fluorophores with structural similarity but varied number of hydrophilic groups, Berezin *et al.* found that more hydrophilic dyes exhibited up to 2 orders of magnitude lower binding constants toward albumin than their hydrophobic counterparts 34. Cypate possess a stronger binding constant to albumin with a value of 556000 M^-1^, whereas IRDye800 binds the weakest with a value of 6100 M^-1^ 34. Though it is difficult to determine the non-covalent label's physico-chemical mechanisms (hydrophobic interactions, electrostatic interactions, and hydrogen bonding), structural modification to the aromatic moiety exhibited slight to moderate changes to the hydrophobic nature of the dyes. As a result, the BSA binding constant could be referred as a measure for hydrophilicity 39. In addition, it is well-established that aromatic groups of the dyes primarily mediate albumin binding 40,41. The accessibility of the dye's chromophore core to the conjugate system of BSA, which was affected by the length of sulfonate groups and conjugation, plays a key role in albumin binding. As reported by Beckford, *et al.* 42*,* increased hydrophobicity of the indolium side chain results in enhanced binding interaction. In the specific case of Cy7.5 and IRDye800, the varied chromophore accessibility was the combined results of steric hindrance and electrostatic repulsion caused by the phenyl groups and sulfonate groups. Accordingly, our study demonstrated that HA-Cy7.5 displayed increased binding with BSA as compared to HA-IRDye800 (**Figure 2B-C**), which could be due to the benzoindole of Cy7.5 being more hydrophobic than the IRDye800 indole. Also, ionized sulfonate groups significantly decrease the hydrophobicity of IRDye800 and in turn decrease serum protein binding.

### Background Signal Interference can be Optimized to Detect Pancreatic Cancer

To investigate the hypothesis that protein association resulting from dye hydrophobicity/hydrophilicity and HA MW_N_ could affect the biodistribution profile, each conjugate, or dye alone, was intravenously injected into wild type (WT) C57BL/6 mice or PDAC-bearing mice (age and gender matched), and then necropsied at either 24 or 96 h post injection. After necropsy, the fluorescence intensity was examined in each organ for both HA-Cy7.5, HA-IRDye800 and control groups. In the WT mice, the organs of interest, RES organs (liver, spleen and femur) and clearance organs (liver and kidney), were compared between different MW_N_ of HA-dye for NIRF accumulation. HA-Cy7.5 conjugates primarily accumulated in liver, followed by spleen and kidney, among which, HA_20k_-Cy7.5 displayed the highest fluorescence intensity in liver that lasted for 4 days (**Figure S3A-B, Figure S2A**). As a comparison, HA-IRDye800 interacted slightly with albumin (**Figure 1C, 2C**) and was able to retain the small size and hydrophilicity of HA and IRDye800. Therefore, the resulting biodistribution profile was attributed to the HA-IRDye800 conjugate rather than albumin (**Figure S3C-D**). When comparing different MW_N_ of HA-IRDye800, HA_5k_-IRDye800 and HA_20k_-IRDye800 exhibited greater tendency to be accumulated and cleared in kidneys as compared to liver (**Figure S3C-D,** Video 2), whereas HA_100k_-IRDye800 appeared to be retained in RES organs.

**Figure 3:**
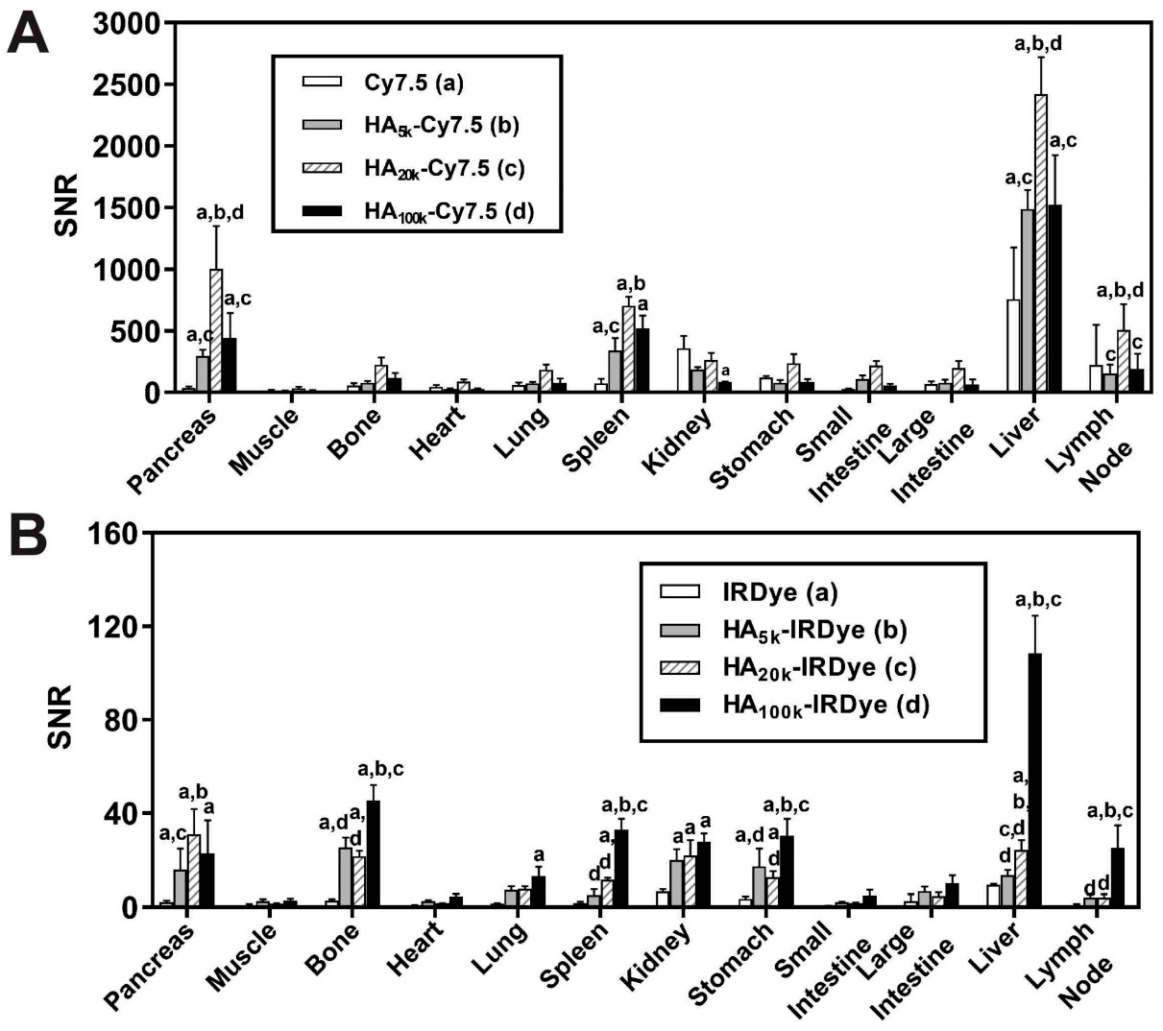
Quantification of biodistribution and the ratio of tumor contrast to accumulation/clearance organs from PDAC-bearing mice treated with HA-dye conjugates or controls 24 h post-administration. Relative organ biodistribution of (**A**) HA-Cy7.5 and (**B**) HA-IRDye800. Quantification was based on fluorescence intensity from NIRF images; N = 5, ^a^*p* < 0.05 as compared to free dye, ^b^*p* < 0.05 as compared to HA_5k_-dye, ^c^*p* < 0.05 as compared to HA_20k_-dye, ^d^*p* < 0.05 as compared to HA_100k_-dye.

The biodistribution pattern of non-pancreatic tissue was comparable between WT C57BL/6 mice (**Figure S3A, C**) and the PDAC mouse model (**Figure 3A-B**). In the PDAC-bearing mice, higher fluorescence signal was observed in pancreas from the HA-dye-administered group compared with the free dye group (*p* < 0.0001). The fold-increase of NIRF signal of the pancreas was 8-28 for HA-Cy7.5 when compared to Cy7.5; and 8-15 for HA-IRDye800 when compared with IRDye800 (**Figure 3A-B**).

Background organ interference ratios were quantified by dividing the fluorescent intensity of PDAC by that of visceral organs in the PDAC-bearing mice. These organs include muscle (reference tissue for non-NIRF accumulation), stomach, small intestine, liver, spleen and kidneys (surrounding organs or clearance organs). The ratios are summarized in **Table S2-3**. The ratio for PDAC: muscle was higher for HA-conjugated dye compared to the free dyes, and the ratio remained ≥10 after 96 h clearance (calculated from **Figure S4B, D, S5I**), thus presenting high PDAC specificity. The impact of clearance routes on tumor contrast was determined by quantifying the ratios of PDAC:clearance organs. The ratio for kidneys was 4.7 compared to 2.0 and for liver it was 0.5 compared to 1.8, for HA_20k_-Cy7.5 and HA_20k_-IRDye800 respectively (**Table S2**). The values indicate that the largest source of NIRF signal interference for the HA-Cy7.5 conjugates were the liver and spleen, while in HA-IRDye800 it was the kidneys.

An ideal molecular imaging agent should display suitable pharmacokinetics for visualizing the biochemical target/process of interest. Insights have been reported into the relationship between molecular structure and biologic behavior of cyanine dyes that's determined by the protein association 34,39,41,43,44. Tian *et al.*^41^ improved the pharmacokinetics with albumin-chaperoned cyanine dyes, the complex afforded impressive blood vessel resolution and expanded time window, when compared to free cyanine dyes. The dye-protein complexation strategy can be extended to antibodies for molecular targeted cancer imaging 41. On the other hand, physiological parameters such as hepatic filtration, tissue extravasation, tissue diffusion, and kidney excretion are clearly impacted by imaging agent size, which has a profound impact on its *in vivo* distribution 45,46. Often macromolecules (10 nm - 100 nm 45) afford prolonged blood half-lives to allow time for extravasation out of the vasculature. Nevertheless, small particles exhibit lower background signal due to rapid clearance, but provided less time for the probe to access the tumor 47. As a linear homopolymer, HA demonstrated MW-dependent-biodistribution 48. Courel, *et al.* 49 found the retention of tritiated high MW (HMW)-HA was 40-fold higher than that of HA oligomers on nude mice 5 h post injection; in addition, the highest accumulation was in kidney for HA oligomers and in liver for HMW-HA. Though conjugated with NIR fluorophore, HA-IRDye800 demonstrated consistent MW-dependent biodistribution as HA (**Figure S3C-D**) because HA MW drives the *in vivo* fate instead of the hydrophilic fluorophore. The Cy7.5 moiety entropically favors albumin binding (**Figure 1B, 2B**). Therefore, the increased size of the HA-Cy7.5 + albumin complex was excluded from renal filtration and entrapped and subsequently eliminated through hepatic filtration (**Figure S3A-B**).

### FIGS can Detect Pancreatic Adenocarcinoma

**Figure 4:**
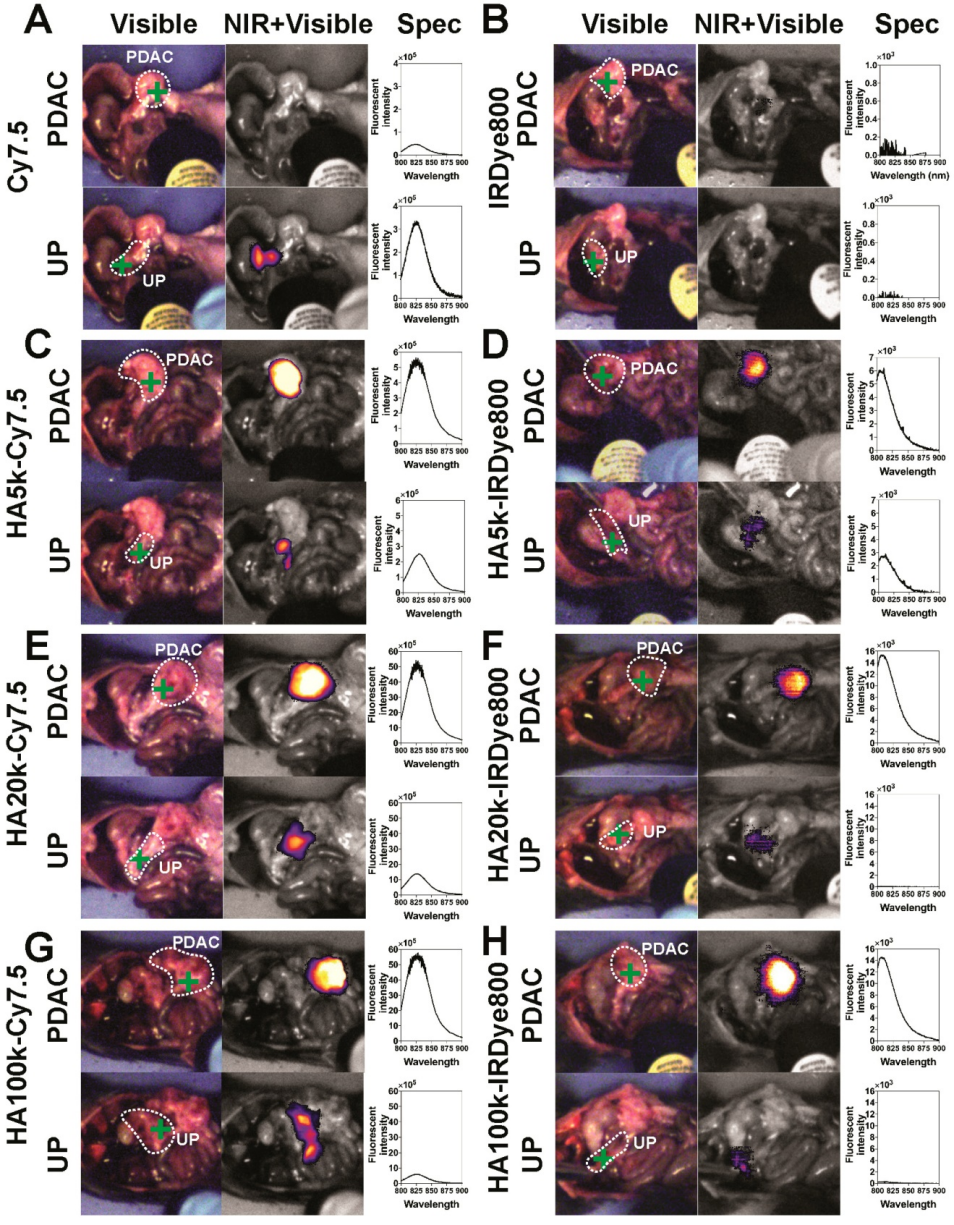
Images from optical surgical navigation of PDAC with contrast enhancement due to HA-dye or dye 24 h post *i.v.* injection. Livers and spleens were removed due to high background signal due to clearance, which interferes with pointed spectroscopic measurement. Spleens that were completely involved with PDAC were kept. Two channels (visible with/without NIR) and two spots [Spectropen directed at PDAC, uninvolved pancreas (UP)] were displayed for each experimental group: (*Left*) Color images depict orthotopic PDAC and the location of the handheld spectroscopic pen, which provides NIR spectral information and serves as an excitation source for a widefield imaging system; white dots outlined PDAC or UP. (*Middle*) NIR signal overlaid onto the grayscale image shows robust enhancement of syngeneic, orthotopic pancreatic cancer, green crosses indicate the directed location of excitation laser. (*Right*) Spectroscopic measurements quantifying the fluorescence intensity emitted from PDAC or UP. Improved contrast can be observed in HA conjugated dye groups. UP = uninvolved pancreas.

The representative FIGS images from HA-dyes displayed marked contrast between PDAC and healthy, uninvolved pancreas (**Figure 4**). Overall, HA-Cy7.5 exhibited higher fluorescence intensity than HA-IRDye800, including significant signal in the GI tract, especially for HA_20k_-Cy7.5. In comparison, HA-IRDye800 displayed comparable or greater contrast within the tumor-bearing pancreas along with negligible background signal from the GI tract. The fold increase of signal intensity when the excitation source is directed at the tumor compared to when the excitation source is directed off the tumor is 0.14 (Cy7.5), 2.10 (HA_5k_-Cy7.5), 3.59 (HA_20k_-Cy7.5), 9.17 (HA_100k_-Cy7.5), as detected at 825 nm, and 0 (IRDye800), 2.17 (HA_5k_-IRDye800), 663.09 (HA_20k_-IRDye800), 52.32 (HA_100k_-IRDye800), as detected at 810 nm. The center of the directed excitation laser is indicated by green crosshairs. PDAC and UP were delineated with white dots. The PDAC specificity was maintained for up to 4 days post injection with HA-Cy7.5, but contrast was compromised over time for HA-IRDye800, especially for HA_20k_ and HA_100k_ (**Figure S5**) at 96 h.

The hand-held spectroscopic imaging device utilizes a NIR laser diode (emitting at 785 nm) coupled to a compact head unit for light excitation and collection. Its ability to resolve NIRF signals from background signal arises from the optical filtering that takes place in the hand-held pen. A dichroic mirror and a long pass filter attenuate Rayleigh scattering by a factor of 10^8^ in the collection fiber, thus, only Stokes-shifted light is transmitted to the spectrometer 50. The Spectropen is highly sensitive for measuring exogenous NIRF contrast agents during surgical procedures and it stably aligned and calibrated for robust surgical use, with the minimum spectrally-resolvable concentration of ICG at 2-5 ×10^-11^ M in the *in vitro* setting 50. As compared to the imaging systems that relied on native fluorescence, incorporation of contrast agents into spectroscopic imaging also enables addressing the issue of tumor heterogeneity. In this study, the signal from UP in **Figure 4** might be interfered from intestinal tissue that is immediately beneath the UP. The UP signal could also be the result of marginal distribution of the relatively brighter Cy7.5-based contrast agents. The signal in the heatmap is obtained solely from the excitation area of the laser, indicated by green crosshairs in **Figure 4**.

Tumor contrast enhancement relies not only on the accumulation of the contrast agent in tumor tissue, but also on the ability of the contrast agent to be cleared from healthy surrounding tissues and organs. Accumulation and clearance of imaging agent from tissue that is not malignant is the sum of several factors, including native dye clearance mechanism, physico-chemical properties of the contrast agent, and/or for potential phagocytic interaction, which directs agents to organs of RES 51,52. Therefore, there are several important criteria that can be tuned to control the *in vivo* biodistribution of contrast agents 46. Size and hydrophobicity are decisive factors for kidney elimination and RES sequestration 53: generally, particles with a hydrodynamic diameter up to 5-7 nm fall below renal filtration threshold and are excreted 54, while particles larger than 200 nm can be trapped in liver and spleen with prolonged retention. Hydrophilicity variation affects the pharmacokinetics of the probes as a result of distinct protein adsorption, which is more likely for hydrophobic than hydrophilic materials 55. Protein adsorption could mediate the enhanced RES sequestration by promoting opsonization 55. More importantly, serum protein adsorption increases the apparent hydrodynamic diameter of a hydrophobic molecules by more than 15 nm, which prevents renal excretion 19. The optimum pharmacological properties for *in vivo* application include minimum non-specific binding and an adequate retention time in the body preferably followed by fast excretion 56. A better understanding with regard to how the characteristics of contrast agents influence their *in vivo* behavior is an important step towards designing NIRF biomaterials suitable for molecular imaging applications and for efficient tumor delivery 52.

### Ex Vivo Analyses of NIRF Contrast in PDAC-bearing Pancreata

**Figure 5:**
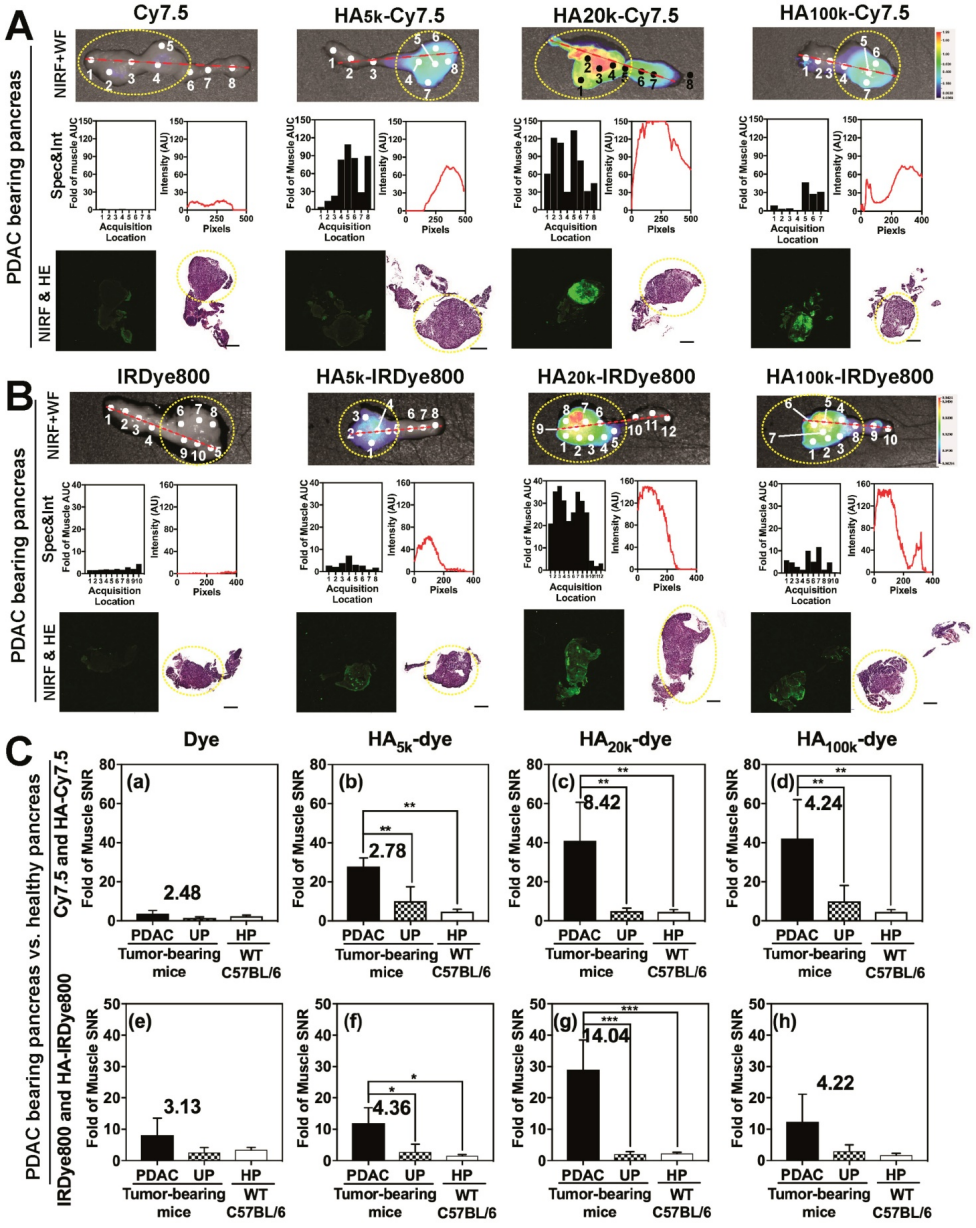
*Ex vivo* analysis of HA-dye accumulation within the pancreases from tumor-bearing mice. Panel **A-B** were arranged into three rows for each group: (*Top*) NIRF and white-field (WF) images of a representative PDAC-bearing pancreas marked with acquisition locations of the spectroscopic signal shown in *middle row*. Yellow dashed lines circle indicated the PDAC portion of pancreas.; (*middle, left*) fluorescence intensity excited with 80 mW laser power and 1 s integration time with the excitation laser component of FIGS system; (*middle, right*) plots of intensity values along the red dashed line from the NIRF images quantified by ImageJ; (*bottom, left*) scanned image of frozen-sectioned unstained healthy/PDAC-bearing pancreas with NIR channel from Odyssey Clx imaging system; (*bottom, right*) scanned H&E stained pancreas slides with Pannoramic 250 flash series digital scanner, for each group pancreases were sequentially sectioned with unstained slides. Yellow dashed lines circle indicated the PDAC portion of pancreas. All scale bars represent 2 mm; (**C**) SNR of PDAC, uninvolved pancreas (UP) and healthy pancreas (HP) normalized by muscle SNR that's been treated with (a) Cy7.5, (b) HA_5k_-Cy7.5, (c) HA_20k_-Cy7.5, (d) HA_100k_-Cy7.5, (e) IRDye800, (f) HA_5k_-IRDye800, (g) HA_20k_-IRDye800, (h) HA_100k_-IRDye800. Contrast data was obtained 24 h after *i.v*. injection. SNR values were calculated from NIRF images obtained with the Pearl Trilogy Small Animal Imaging System. ***p < 0.001, **p < 0.01, *p < 0.05, N = 4-5 for each group.

As observed in **Figure 5** and**Figure S6**, NIRF images (*Top row*) of the pancreas indicated that HA_20k_-NIRF conjugates did not accumulate in the healthy pancreas (HP) of WT mice (**Figure S6**), or uninvolved pancreas (UP) of PDAC tumor-bearing mice (**Figure 5A-B**), but accumulated in the PDAC portion of the pancreas, thus indicating an enhanced PDAC specificity. Additionally, HA_5k_- and HA_100k_-NIRF conjugates also specifically-accumulated in the pancreatic tumor, but with lower intensity than HA_20k_-NIRF conjugates. The NIRF intensity from the Spectropen at acquisition locations across the pancreas is consistent with the NIRF intensity profiles of the tumor-bearing pancreata imaged with LI-COR imaging system (**Figure 5A-B**). A board-certified pathologist (GAT) identified tumor with histopathology and areas with high NIRF signal were also areas identified as poorly differentiated PDAC (**Figure 5A-B**), which is well-perfused by HA_20k_-NIRF as compared with HA_5k_-NIRF and HA_100k_-NIRF. Signal was not detected in free dye-treated PDAC or healthy pancreas. Contrast was still visible after 4 days of clearance (**Figure S7**), especially for the 100k HA conjugates. In summary, HA conjugated dyes are effective at the specific identification of PDAC, with HA_20_ being the most efficient vehicle for PDAC delivery.

To investigate the relationship between potential HA-CD44 targeting and PDAC contrast-enhancement, both *KPC* (LSL-Kras^G12D/+^; LSL-Trp53^R172H/+^; Pdx-1Cre) mice-derived PDAC cells and resected tumor specimens were analyzed for CD44 expression (**Figure S8**). Additionally, single-stained flow cytometry (FC) histogram demonstrates that KPC cells were positive for CD44, (**Figure S8A**) with a stain index of 12.45 AU compared to 2.94 AU or primary pancreatic epithelial (PPE) cells (Quantified from FC). The necropsied tumors were composed of markedly atypical, non-cohesive epithelioid cells with marked nuclear pleomorphism and numerous mitotic figures. In some areas, the tumor cells demonstrated a spindle cell morphology suggestive of sarcomatoid differentiation. CD44 staining revealed strong, diffuse membrane staining in all tumor cells with both appearances (**Figure S8B**).

To compare the contrast enhancement of HA-dye statistically and quantitatively, SNR of PDAC was divided by that of uninvolved pancreas or healthy pancreas from WT mice. As shown in **Figure 5C**, no significant difference of NIRF signal was detected between uninvolved pancreas and healthy pancreas, regardless of contrast agent treatment. In comparison, a significantly-higher signal was detected between PDAC and uninvolved pancreas for HA_5k_-, HA_20k_- and HA_100k_-Cy7.5, HA_5k_- and HA_20k_-IRDye800 but not HA_100k_-IRDye800 treated mice, the contrast ratio (defined by SNR of PDAC/SNR of UP) was labeled in each figure. HA_20k_-IRDye800 displayed the highest fold-increase of 14.0, with *p* < 0.001. HA-Cy7.5 exhibited increased contrast as compared to Cy7.5, regardless of MW_N_ of HA. Furthermore, enhanced contrast within PDAC pancreas was detectable after 4 days, with a contrast ratio of 3.86 and 5.96 for HA_20k_-Cy7.5 and HA_20k_-IRDye800, respectively (**Figure S7I**).

The tumoral retention of HA-dye depends on the interstitial binding of HA moiety after blood circulation and extravasation. PDAC is hypovascular with collapsed, but intact blood vessels, which is not desirable for EPR effect 57-59. Cabral, *et al.* 60 reported that sub-30 nm micelles could penetrate vasculature of pancreatic tumor. The calculated experimental hydrodynamic diameter of 30 kDa HA is 15 nm, while 32 nm for 100 kDa HA 61. Therefore, HA_5k_, HA_20k_ conjugated dye are presumed to extravasate more easily than HA_100k_-dyes, meanwhile vascular bursts could potentially enhance permeability of pancreatic tumor blood vessels for large particles 62. Additionally, PDAC is characterized with an extensive extracellular HA deposition, which is (34 ± 2.7) ng/mg tissue for normal pancreas in contrast to (420 ± 150) ng/mg for PDAC in *KPC* mice 63-65. A wide variety of HA binding molecules (serum-derived HA-binding protein, versican) and receptors (CD44, LYVE-1, RHAMM) contribute to the formation of HA meshwork and anchorage to the cell surface 64. Followed with extravasation, HA moiety of contrast agents bind to overexpressed CD44 (or potentially other HA binding receptors) (**Figure S8**), which could confine the conjugates in tumor interstitium as opposed to diffusing back into blood vessels. Furthermore, the binding affinity relates with HA MW:HA oligomers with size of 38 disaccharide units (around HA_20k_) showed higher avidity with CD44 compared with that of 10 disaccharide units (around HA_5k_) due to multivalent binding 66. In summary, PDAC vasculature is available for LMW HA-dye's extravasation, and HA-CD44 binding plays a role in retention within tumor, which may be the mechanism of HA-dye's robust contrast in PDAC.

### Contrast Enhancement for the Detection of Primary PDAC and Intraperitoneal Metastases with Widefield Imaging

**Figure 6:**
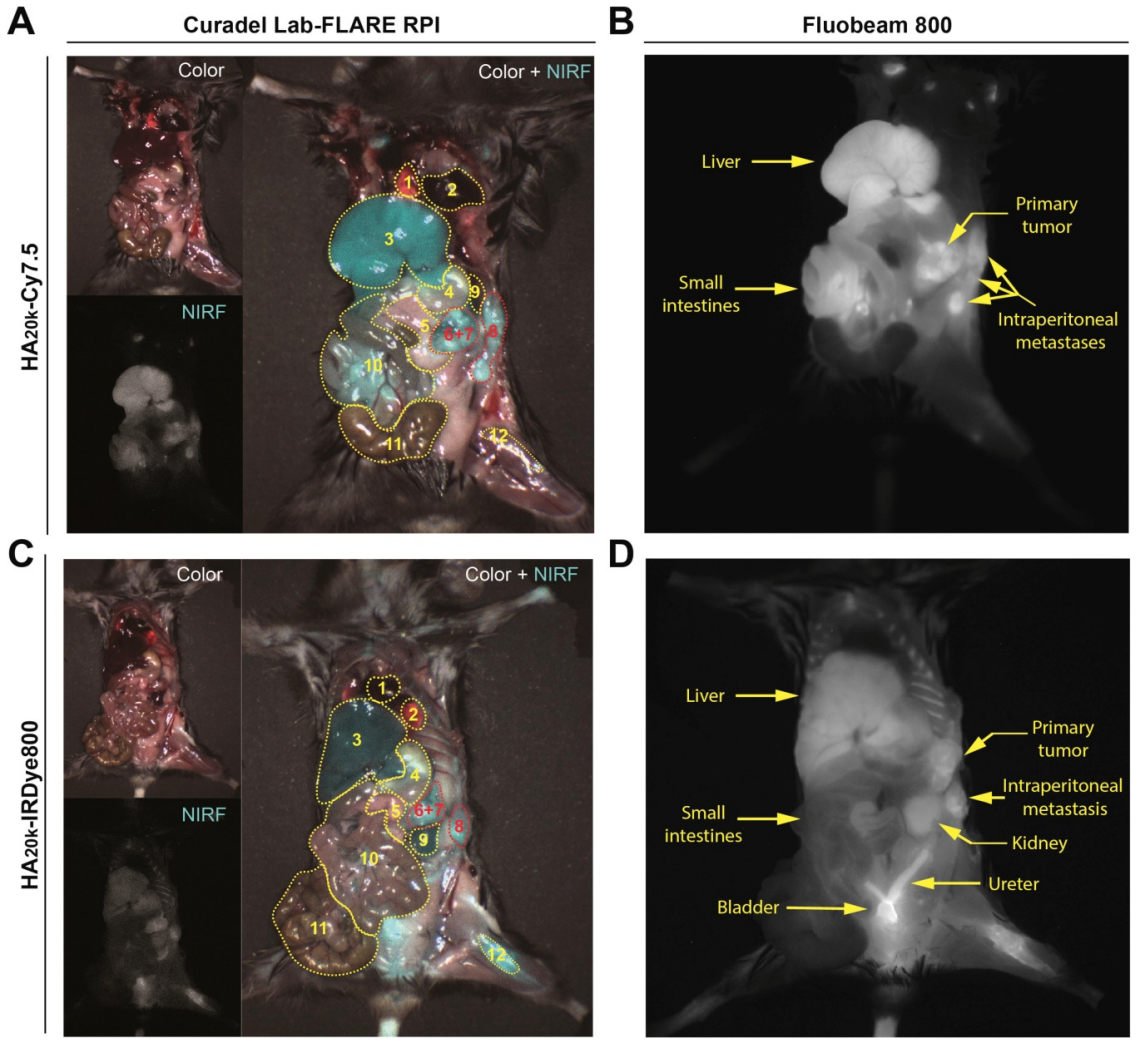
Representative whole-body images of PDAC-bearing mice captured by the Lab-FLARE RP1 (A, C) or Fluobeam 800 (B, D) imaging system 24 h post injection of contrast agents. Images were captured from the color and NIRF channel and merge channels of the Lab-FLARE RP1 imaging system are shown on the left, while images obtained by the Fluobeam 800 imaging system are shown on the right. The administered dose was 1 nmol of Cy7.5/mouse for HA_20k_-Cy7.5, and 10 nmol IRDye800/mouse of for HA_20k_-IRDye800. 1- Heart, 2- Lung, 3- Liver, 4- Stomach, 5- Uninvolved pancreas, 6+7- Primary pancreatic tumor and involved spleen, 8- Introperitoneal tumor, 9- Kidney, 10- Small intestines, 11- Large intestines, 12- Femur. Yellow dotted lines indicate the organs of interest and are labeled at the bottom of the figure. The intensity (cyan pseudocolor; Curadel Lab-FLARE RPI) and (grayscale colormap; Fluobeam 800) represents the NIRF accumulation. *N* = 3.

We further investigated the role of RES accumulation (HA_20k_-Cy7.5) and kidney accumulation/elimination (HA_20k_-IRDye800) in contributing to the PDAC contrast by employing widefield imaging systems to simultaneously capture the NIRF distribution among organs of interest (**Figure 6, Video 1-3**). Overall both conjugates preferentially accumulate primary PDAC (**Figure 6A, 6C**, organs labeled 6+7), and more interestingly, intraperitoneal metastatic lesions (**Figure 6A, 6C** organ labeled 8) with high contrast. HA_20k_-Cy7.5 (**Figure 5A-B**) exhibited significant accumulation in liver and spleen as compared to HA_20k_-IRDye800 (**Figure 5C-D**), even with one tenth of the dose of dye as compared to HA_20k_-IRDye800. The NIRF signal is evident in liver and small intestines as compared to the kidneys due for HA_20k_-Cy7.5 (**Figure 5A-B**). On the other hand, HA_20k_-IRDye800 (**Figure 5C-D**) is clearly observed in the kidneys, ureters, and bladder, which supports renal elimination of small, hydrophilic HA- conjugates.

Because Cy7.5 is brighter than IRDye800 67 (also demonstrated in the different SNR scaling of axes in **Figure 3**), when injected with the same doses of HA_20k_-NIRF (equivalent to free dye), NIRF signals from HA-Cy7.5 conjugates were saturated on the Lab-FLARE® imaging system, while not detectable for IRDye800-based conjugates. In order to compare the relative organ accumulation between the two contrast agents while keeping the imaging parameters consistent, we adjusted the injection doses of HA_20k_-IRDye800 to 10 nmol of IRDye800 per mouse. We also determined the acute systemic toxicity with the adjusted doses and did not observe HA_20k_-IRDye800 induced toxicity, as shown in **Figures S9-S10**.

Handheld NIR spectrometers have been used for the quantitative analysis of solid pharmaceutical formulation 68. Judy *et al.*^69^ compared digital imaging with spectroscopy in quantifying fluorescent signal-to-background ratios *in vitro*, murine xenografts, tissue phantoms and clinically, concluding that spectroscopy was the most sensitive in identifying small numbers of cells, and tumors in deep tissue. Even though spectroscopic imaging has superior resolution than digital imaging, it suffers from a limited field of view and was time-consuming for data processing. While digital imaging was the most practical considering its wide field of view, background noise filtering capability and sensitivity to increasing depth, making it useful for broad exploration of the wound and body cavity. The detection threshold of ICG for digital imaging is 10 times higher than that of spectroscopy. In present study, the dose of IRDye800 for whole body fluorescence imaging was 10 times higher than that of Spectropen to achieve comparable signal and contrast, demonstrating lower sensitivity of fluorescence with whole-body fluorescence imaging. Considering the pros and cons for each imaging instrumentation, a useful strategy would be to use digital imaging to scan large regions of body cavity and the wound, and to use spectroscopic device for more detailed analysis of margins and micrometastases.

**Figure 7:**
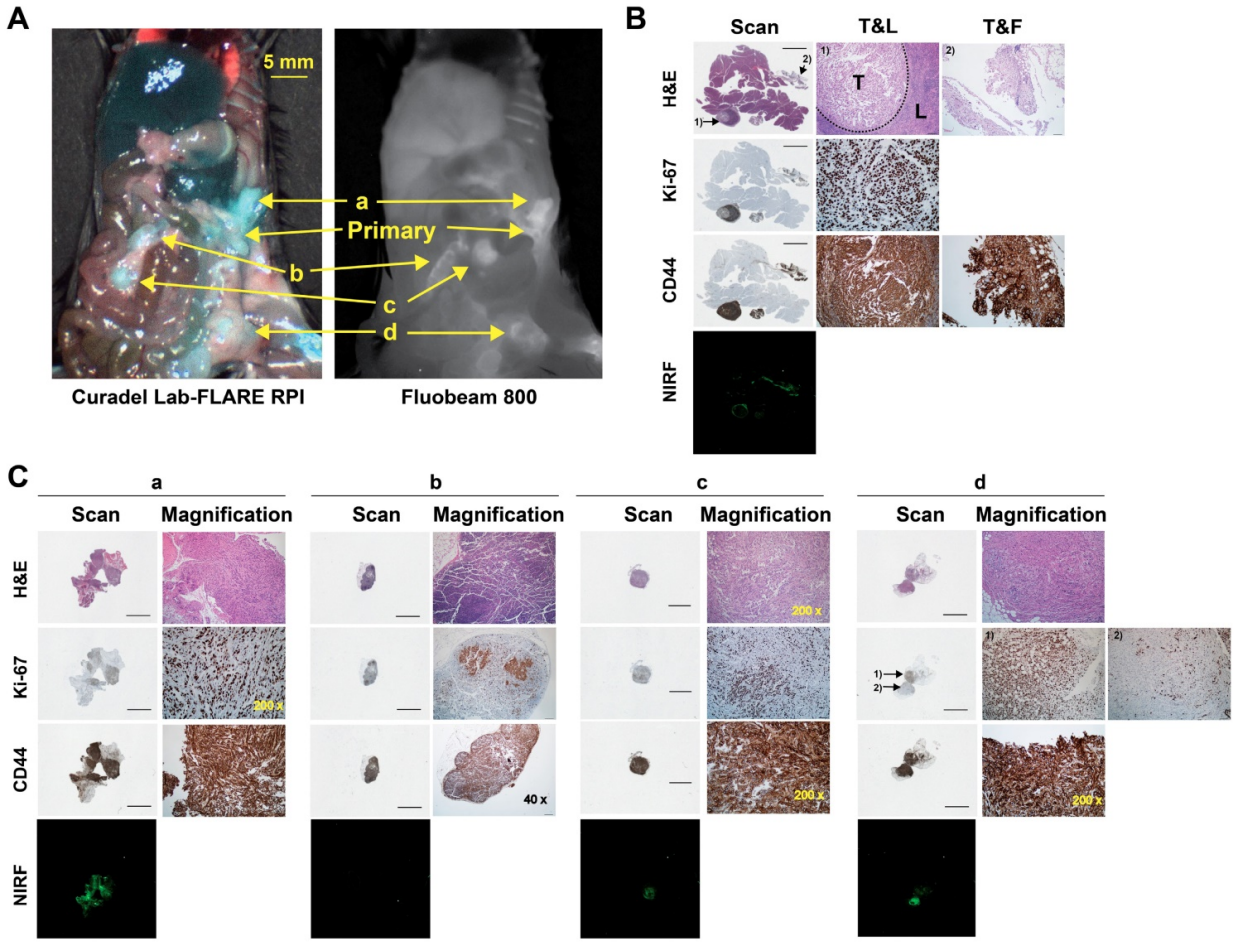
Probing capability of HA_20k_-IRDye800 on the intraperitoneal metastases of PDAC-bearing mice. (**A**) Fluorescence was accumulated at the metastatic sites of interest confirmed by the two wide-field imaging systems; a, b, c, d pointed at the suspected sites of metastases. (**B**) Pathology of primary tumor that was involved with spleen identified with H&E, Ki-67, and CD44 staining, NIRF distribution of corresponding sectioned was determined with 42 μm resolution. The intensity of 800 nm channel of Odyssey Clx imaging system was set at 9. Black bars represent 2 mm on the scanned images. T&L refers to tumor and lymphatic tissue, T&F refers to tumor and fat. 1) and 2) are 200 x magnification of the site pointed at the scanned images. (**C**) Scanned and magnified images with power labeled at right corner. 1) and 2) in d refers to the well differentiated and poorly differentiated tumor, respectively.

HA_20k_-NIRF conjugates were able identify abdominal lesions smaller than 7 mm^3^ and lymphatic metastases (**Figure 7**, **Video 3)**. The presence of malignant tissue was supported by the hematoxylin and eosin (H&E) and positive staining of Ki-67 (**Figure 7B-C**). To that end, detailed histopathology investigation confirmed contrast enhancement of several primary and metastatic PDAC malignances. Sections of the primary lesion demonstrate nodules of epithelioid tumor cells within a peri-pancreatic lymph node and fat, the latter associated with a prominent inflammatory response (**Figure 7B**). The tumor cells demonstrated marked cytologic atypia without definitive glandular formation and adjacent pancreatic parenchyma was unremarkable. Immunostaining revealed that the tumor cells showed strong, diffuse membranous staining for CD44 in the background of expected staining within lymphocytes. While Ki-67 labeled >90% of tumor nuclei. PDAC invasion into soft tissue immediately adjacent to the pancreas had some areas that were more poorly differentiated with foci having a sarcomatoid appearance (**Figure 7C-a**). CD44 stained all tumor cells and Ki-67 labeled approximately 75% of all tumor nuclei. PDAC metastases to adipocytic tissue (**Figure 7C-c**) had consistent morphology and showed diffuse CD44 and strong cytoplasmic staining. Ki-67 labels 50-60% of tumor nuclei. In **Figure 7C-d**, tumor cells are seen involving adipose tissue, surrounding individual adipocytes. Interestingly, there is heterogeneity of morphology with some tumor cells being epithelioid with others having a spindled, sarcomatoid appearance. CD44 strongly labels all tumor cells, which could play a role in HA targeting of these malignancies.

Up-regulation of CD44 correlated with distant metastases and aggressive malignant behaviors of pancreatic cancer 70-72. The regulation of tumorigenesis and cancer metastasis by CD44 was reported to be partially *via* PI3K/AKT or MAPK/ERK regulatory pathway 72. Specifically, PDAC cells expressing high levels of CD44s with a mesenchymal-like phenotype were highly invasive and developed gemcitabine resistance *in vivo* 70. CD44 is also reported to be a cancer stem cell marker for pancreatic cancer cells 73,74. In clinical setting, CD44 expression could help identify patients at high risk of early recurrence and provide diagnostic value for more aggregative treatment after radical surgery 75. Hence, targeting CD44^high^-expressing cells may provide diagnostic/therapeutic strategy for improving survival. In this study, we use CD44 as the malignant biomarker, which has been confirmed to be specifically and overly-expressed in the tumoral cells/samples (Figure S8). Though Ki67 is not a specific marker for PDAC, while subculturing KPC-derived cancer cells, we found the population doubling time is less than 24 h, which in in accordance with Torres *et al.*'s finding that doubling time of KPC-derived cells could be as short as 33 h, as compared with Pan02 (56 h) and AsPC-1 (58 h) 76,77. On the contrary, the primary pancreatic epithelial cells from C57BL/6 mouse have the doubling time around 1.5-2.5 days. Due to the relatively high proliferative efficiency, Ki67 was used to differentiate KPC cells from benign cells as well.

## Conclusion

The results of this study demonstrate that contrast agent biodistribution can be controlled to specific clearance organs while maintaining strong tumor CNR. In particular, SNR was tuned to minimize the signal from liver, spleen, and GI tract. Overall, HA-dye conjugates provide a straight-forward, but versatile platform to intraoperatively image pancreatic cancer. The strategy to reroute the native clearance of cyanine dyes from hepatobiliary to renal elimination and ease the RES burden of macromolecules results in the finding of optimized contrast agent (HA_20k_-IRDye800) with minimal non-specific accumulation in background organs. For conjugates using IRDye800, the dominant factor was HA MW. As shown with the HA-Cy7.5 conjugates, protein association with Cy7.5 normalizes biodistribution to the RES regardless of HA MW. On the contrary, LMW HA-IRDye800 conjugates (5 and 20 kDa HA) are likely below the renal threshold resulting in reduced RES accumulation. This is due in part to the HA MW, but also the lower propensity of IRDye800 to bind serum proteins compared to Cy7.5, as even HA_5k_-Cy7.5 is rerouted to RES accumulation and/or hepatobiliary circulation. Consequently, further detailed studies are ongoing to evaluate specific protein bound populations with these conjugates, pharmacokinetic profiles, and long-term biodistribution and clearance profiles of the lead HA conjugates. The rationale approach reported here control biodistribution could be applied to other delivery systems to minimize RES organ toxicity of therapeutic agents. Alternatively, directing imaging agents away from the kidney would benefit those with renal failure, where administration of certain renally clearing probes is contraindicated.

## Methods

*Materials and Reagents*: Cy7.5 amine was purchased from Lumiprobe (Hallandale Beach, FL); IRDye800CW trifluoroethylamine was purchased from LI-COR (Lincoln, NE); 5 kDa, 20-30 kDa, and 100 kDa sodium hyaluronate were purchased from Lifecore Biomedical (Chaska, MN). Lyophilized powder of BSA was purchased from Sigma (St Louis, MO). Sephadex G-25 PD-10 desalting column was purchased from GE Healthcare (Pittsburgh, PA); KPC cells were obtained from the diseased pancreas of *KPC* mice which were shared by Dr. Hollingsworth. 10-week-old female C57BL/6J mice were purchased from Jackson Laboratories (Bar Harbor, ME). 5.0 chromic gut and 5.0 nylon surgical sutures were purchased from Johnson & Johnson (Somerville, NJ). FITC-labeled CD44 antibody, purified rat mouse BD Fc blocker, propidium iodide (PI) staining solution and FC staining buffer was purchased from BD Bioscience (San Jose, CA). All other chemicals were purchased from Fisher Scientific and used at analytical grade.

*Conjugation and characterization of Cy7.5 or IRDye800 HA Conjugates*: Sodium hyaluronate (3.3-6.9 mg, MW_N_ = 5 kDa; 9.5-10.7 mg, MW_N_ =20-30 kDa; 10.2-12.7 mg, MW_N_ =100 kDa) was completely dissolved in 2 mL of water. EDC and NHS (both 10× molar ratio to amine group), were then dissolved in the HA solution. After 15 min of activation, the pH was raised to 7.2, and Cy7.5-NH_2_ (dissolved in DMSO) or IRDye800-NH_2_ (dissolved in ultrapure water) -at 3× molar ratio to disaccharide unit per HA_5k_ and HA_20k_ chain and 9× for HA_100k_ chain - was each added dropwise to each of the activated HA solutions under constant stirring. The reaction was allowed to proceed for 24 h at room temperature (rt). The reaction contents were then transferred to dialysis tubing (MWCO = 3500 Da for HA_20k_- and HA_100k_-dye conjugates; MWCO = 1000 Da for HA_5k_-dye conjugates, Spectrum Laboratories, Rancho Dominguez, CA). HA-Cy7.5 conjugates were dialyzed against 1:1 EtOH:H_2_O for 24 h followed by H_2_O for an additional 48 h. HA-IRDye800 conjugates were dialyzed against H_2_O for 24 h. The HA-dye conjugates were removed from the dialysis tubing, purified by PD-10 column, lyophilized, and stored at -20 °C. Dye content of HA-dye conjugates was determined relative to dye solutions using absorbance and fluorescence spectroscopy. Specifically, an Evolution 220 spectrophotometer (Thermo Fisher Scientific, Madison, WI) was used for scanning absorbance spectra (600-900 nm) and a FluoroMax-4 spectrofluorometer (Horiba, Edison, NJ) was used for quantifying fluorescent intensity of dye, HA-dye, disassembled and BSA-associated HA-dye from 770 to 900 nm. BSA solution was prepared at 0.33 mg/mL (5 μM) and HA-dye conjugates were prepared at 4 μM of free dye for **Figure 1B-C** and **Figure S2-3**. FEI Tecnai G2 Spirit transmission electron microscope (Hillsboro, OR) equipped with AMT digital imaging system were employed for TEM imaging.

*Determination of nonspecific serum protein binding***:** BSA (50 μM, Sigma-Aldrich, MO) was incubated with HA_20k_-Cy7.5/IRDye800 (4 μM dye equivalent) in phosphate buffer (0.01 M, pH 7.4) for 1 h at rt. 1 mL of mixture or corresponding control (HA-dye conjugate only or BSA only) was filtered through 0.22 μm filter (Fisher Scientific, Hampton, NH) before loading onto an AKTA Pure 25 L Chromatography system (GE Healthcare, Sweden) that was equipped with Superdex200 Increase 10/300 GL column, UV monitor (fixed wavelength at 280 nm), and a fraction collector. For kinetic fluorescence measurements, an outlet portal was connected to a micro cuvette (Starna, Atascadero, CA) for monitoring the fluorescence of the eluent using the fluorescence spectrophotometer. Sample was eluted at flow rate of 0.45 ml/min for 60 min. Kinetic monitoring of fluorescence was collected for 66.7 min with 5 s integration time for HA_20k_-Cy7.5 (slit width was 20 nm, λ_ex_ = 775 nm, λ_em_ = 820 nm) and HA_20k_-IRDye800 (slit width was 10 nm, λ_ex_ = 770 nm, λ_em_ = 790 nm).

*Tumor model induction*: All animal studies were performed under a protocol approved by the UNMC Institutional Animal Care and Use Committee. Procedures were followed in accordance with institutional guidelines per the Guidelines on the Care and Use of Animals for Scientific Purposes to ensure humane care of the animals. Orthotopic, syngeneic PDAC induction was performed as described in previous studies 28,78. Briefly, mature female C57BL/6 mice were selected for orthotopic tumor challenge using 10,000 KPC cells per mouse. Once anesthetized, a ~5 mm incision was made into the skin and peritoneum on the abdomen between the hip and rib. This allowed for the spleen to be exteriorized for injection of PBS cell suspension into the body of the pancreas. The peritoneum and skin were then secured using an internal dissolving chromic gut and a 5.0 nylon surgical suture, respectively. Animals were warmed, hydrated, and monitored until consciousness was regained. Within two weeks of introduction of tumor cells, palpable tumors were detected in all challenged mice.

*Biodistribution of HA-Dye conjugates as measured by fluorescence*: Dye (1 nmol per mouse) or HA-dye (1 nmol dye/mouse equivalent) in 80 μL ultrapure water was intravenously injected into WT C57BL/6 mice or PDAC-bearing mice via tail vein (N = 5 mice/group). Mice were euthanized 24 h or 96 h post injection. WT mice were completely necropsied to examine overall fluorescence distribution of dye or dye conjugates. Dissected organs were imaged on a Pearl Trilogy small animal imaging system (LI-COR Biosciences, Lincoln, NE). The fluorescence intensity of each organ was collected with the 800 nm channel and analyzed with Image Studio Ver. 5.0 software (LI-COR Biosciences, Lincoln, NE). The periphery of each organ was identified by manually-defining the region of interest (ROI). Average pixel intensity was used to calculate SNR, which is defined by average tissue intensity per pixel in an ROI: standard deviation of background ROI.

*Simulated fluorescence guided imaging of PDAC*: Using the aforementioned dose, time, and contrast agents, mice were administered NIR fluorophore or HA-NIRF dye conjugates. After euthanasia, but prior to intraoperative imaging, the liver and spleen were removed to expose the pancreas and minimize background interference of those contrast agents cleared by the RES. A custom-designed FIGS system was employed to detect contrast enhancement of dye and HA-dye in the pancreatic tumors. The imaging system utilizes a handheld fiber-coupled spectroscopic unit that emits an excitation laser at 785 nm and collects wavelength-resolved NIR emission (DeltaNu; Laramie, WY). The spectroscopic unit also serves as the excitation source for a real-time widefield imaging system (Spectropath; Atlanta, GA, USA) that merges a NIR channel (800-950 nm) and a visible color channel for spatial orientation of the NIR signal. The overall design and integration of these systems has been previously reported 79. A laser power of 80 mW at 785 nm was used for wavelength-resolved (800-950 nm) and widefield imaging.

After FIGS, all mice were necropsied. The pancreas was removed *en bloc* to preserve the anatomical integrity of the primary, stromal, and healthy tissues for fluorescence imaging (using the method described for WT mice) and histological sectioning. To compare against the intraoperative imaging analysis of each pancreas that was obtained during surgery, a straight line was drawn longitudinally across the pancreas in the image obtained by the Pearl Trilogy imaging system. The NIRF intensity along the line was quantified with the *plot profile* function in ImageJ 1.49v software (National Institutes of Health, Bethesda, MD). Tumor-bearing pancreata were directly compared against WT pancreata for all contrast agents.

A simulated surgical procedure was performed to evaluate tumor contrast with RES organs present in the surgical cavity. HA_20k_-NIRF dye conjugates (equivalent to 1 nmol of Cy7.5 or 10 nmol of IRDye800 per mouse) were administered to PDAC-bearing C57BL/6 mice for the acquisition of whole-body images. Mice were euthanized 24 h post injection. Wide-field imaging was acquired with two intraoperative imaging systems. A Lab-FLARE® RP1 small animal imaging system (Curadel, Marlborough, MA) compacted with whole-body excitation source and ≈ 800 nm NIRF emission was employed for the simultaneous determination of fluorescence from PDAC and surrounded abdominal organs. The overall design and operation of this system have been previously reported 80. The Lab-FLARE® imaging system was also utilized to record videos. Exposure time of 150 ms and 12 ms were used for the NIRF laser and white light across all experimental groups, respectively. To confirm the fluorescence mapping, FDA-approved Fluobeam® 800 imaging system (Fluoptics, Cambridge, MA) was employed for whole-body NIRF imaging. The imaging system excites with a 780-nm emission laser and records with a CCD camera with >800 nm emission filtering. The exposure time of the NIRF laser was set to 100 ms and was consistent across all experimental groups.

*Histological analysis*: Tumor tissue was embedded in OCT mounting media gel and was rapidly frozen in liquid nitrogen (**Figure 5**,** S8**) for subsequent staining with H&E or left unstained for NIRF microscopy. Pancreatic tissue was positioned to obtain a maximal footprint for both diseased and healthy pancreases. These samples were cut with a cryostat (Leica Biosciences, Buffalo Grove, IL) at a thickness of 8 μm. For immunohistological staining (**Figure 7**,** S8**), the pancreas was fixed with 4% paraformaldehyde solution for 24 h followed with 70% ethanol. Samples were embedded in wax and sectioned sequentially for immunohistological and H&E staining. Anti-CD44 antibody (Abcam, ab112178, Cambridge, MA) was applied at a dilution of 1:100. Anti-Ki67 antibody (Abcam, ab16667, Cambridge, MA) was applied at a dilution of 1:200. Representative unstained slides were scanned with Odyssey Clx imaging system (LI-COR, Lincoln, NE) with 800 nm channel. Exposure time was consistent among each group (HA-Cy7.5 and HA-IRDye800). H&E-stained slides were scanned with Panoramic 250 flash series digital scanner (3DHistech, Hungary). Representative microscopic photos for IHC and H&E were imaged and captured with an IX73 Inverted Microscope equipped with a DP80 Digital Camera and displayed by CellSens Dimension 1.13 software (all from Olympus, Japan).

*Statistical Analysis*: Data was analyzed with Prism 7 software (Graphpad, La Jolla, CA). Biodistribution and background interference were analyzed using a two-way ANOVA and Dunnett's multiple comparisons test; *ex vivo* analysis of contrast within pancreas was analyzed using one-way ANOVA with Tukey's multiple comparisons test; all data is shown as mean ± standard deviation (SD).

## Supplementary Material

Supplementary figures, tables, and video legends.

Supplementary video 1.

Supplementary video 2.

Supplementary video 3.

## Abbreviations

BSA: bovine serum albumin
CNR: contrast-to-noise ratio
Cy7.5: Cyanine 7.5
DMSO: dimethyl sulfoxide
EDC: 1-Ethyl-3-(3-dimethylaminopropyl)-carbodiimide
FC: flow cytometry
HA: hyaluronic acid
HMW: high molecular weight
HP: healthy pancreas
ICG: indocyanine green
KPC: *KPC* (LSL-Kras^G12D/+^, LSL-Trp53^R172H/+^, Pdx-1Cre)
LMW: low molecular weight
MW_N_: molecular weight
NHS: N-hydroxysuccinimide
NIRF: near infrared fluorescent
PDAC: pancreatic ductal adenocarcinoma
PPE: primary pancreatic epithelial
RES: reticuloendothelial system
ROI: region of interest
SEC: size exclusion chromatography
SNR: signal-to-noise ratio
UP: uninvolved pancreas
WT: wide type
