# Supplementary material for: Tuned near infrared fluorescent hyaluronic acid conjugates for delivery to pancreatic cancer for intraoperative imaging
Source: Theranostics. 2020 Feb 10;10(8):3413–29. doi: 10.7150/thno.40688 (PMC7069077; doi:10.7150/thno.40688)
Supplement: Supplementary file 1 — Supplementary figures, tables, and video legends. [file thnov10p3413s1.pdf]

Tuned near infrared fluorescent hyaluronic acid conjugates for delivery to pancreatic cancer for intraoperative imaging

Bowen Qi,[1] Ayrianne J. Crawford,[2] Nicholas E. Wojtynek,[2] Geoffrey A. Talmon,[3] Michael A. Hollingsworth,[2,4] Quan P. Ly,[4,5] Aaron M. Mohs[1,5,6,*]

[1]Department of Pharmaceutical Sciences, [2]Eppley Institute for Research in Cancer and Allied Diseases, [3]Department of Pathology and Microbiology, [4]Fred and Pamela Buffett Cancer Center, [5]Department of Surgery, and the [6]Department of Biochemistry and Molecular Biology, University of Nebraska Medical Center, Omaha, Nebraska 68198, United States

Running Title: *tuned fluorescent hyaluronic acid conjugates for pancreatic cancer imaging*

Table of Contents

Supplementary Figures

Figure S1. Photochemical characterization of HA-NIRF conjugates in varied solvents.

Figure S2. TEM images of BSA with/without addition of HA_{20k}-dye conjugates.

Figure S3. Biodistribution of HA-NIRF in W.T. C57BL/6 and PDAC-bearing mice.

Figure S4. Images from surgical navigation of pancreatic tumor contrast-enhanced with HA-dye 96 h post *i.v.* injection.

Figure S5. *Ex vivo* analysis of HA_{20k}-dye accumulation in healthy pancreas from W.T. C57BL/6 mice 24 h post injection.

Figure S6. *Ex vivo* analysis of HA-dye accumulation in pancreatic tumor or healthy pancreas from W.T. C57BL/6 mice 96 h post injection.

Figure S7. CD44 expression in PDAC cells *ex vivo* and *in vitro*; *ex vivo* analysis of HA-dye accumulation in pancreatic tumor or healthy pancreas from W.T. C57BL/6 mice 96 h post injection.

Figure S9. Comprehensive biochemical and hematological assessments for WT C57BL/6 mice injected with HA_{20k}-NIRF (equivalent to 20 nmol of free dye/mouse) or vehicle control for the acute toxicology study.

Figure S10. Representative images for the histological assessments of vital organs harvested from WT C57BL/6 mice injected with HA_{20k}-NIRF (equivalent to 20 nmol of free dye/mouse) or vehicle control. $N = 3$.

Supplementary Tables

Table S1. Chemical characteristics of HA-dye conjugates

Table S2. Ratio of HA-Cy7.5-treated pancreas or PDAC fluorescence divided by signal of

surrounding organs 24 h post injection (N = 5)

Table S3. Ratio of HA-IRDye800-treated pancreas or PDAC fluorescence divided by signal of surrounding organs 24 h post injection (N = 5)

Video 1. Wide-field imaging recorded for the NIRF accumulation of HA20k-Cy7.5 in primary tumor and intraperitoneal metastases in PDAC bearing mice.

Video 2. Wide-field imaging recorded for the NIRF accumulation of HA20k-IRDye800 in primary tumor and intraperitoneal metastases in PDAC bearing mice.

Video 3. Wide-field imaging recorded for the NIRF accumulation of HA20k-IRDye800 in abdominal metastases in PDAC bearing mice.

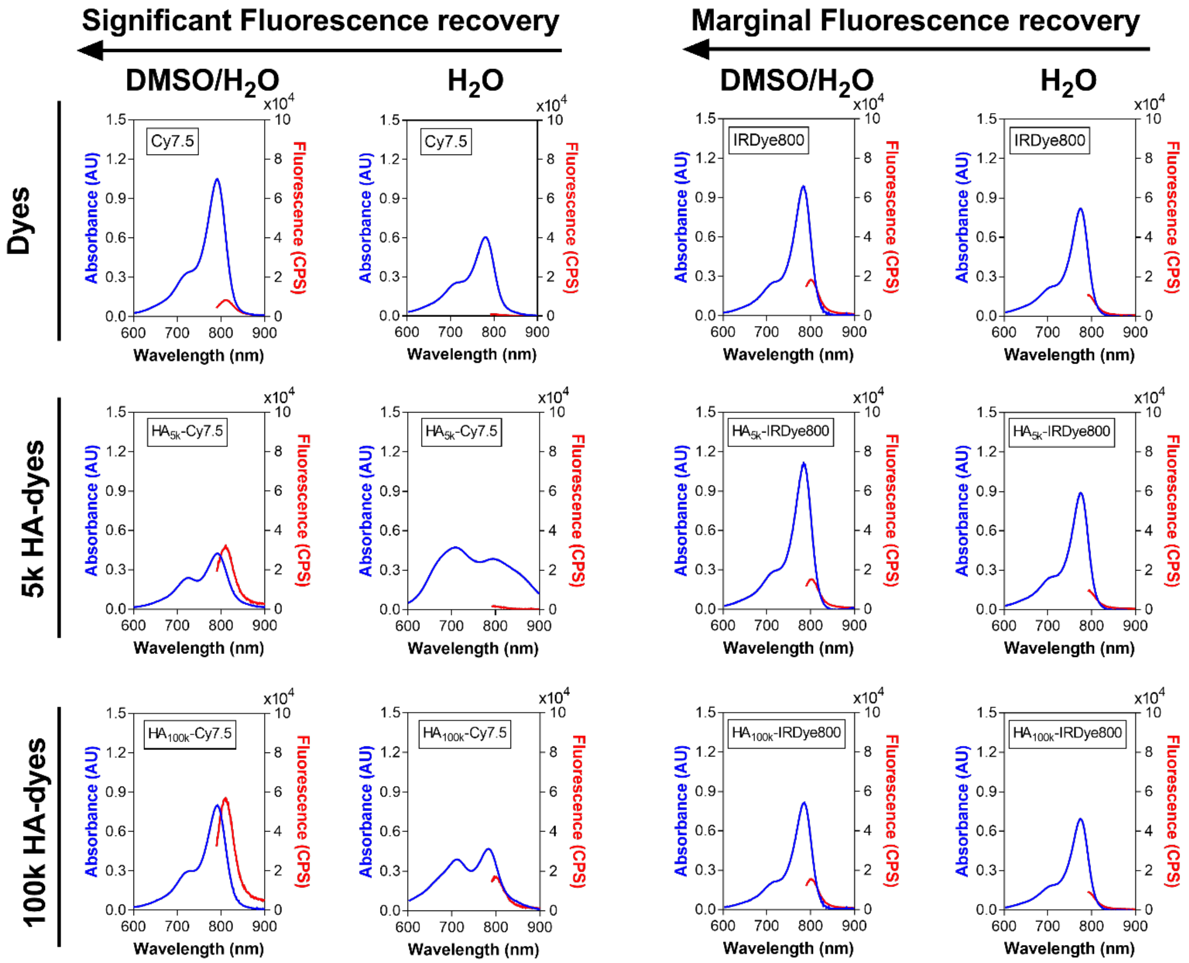

Figure S1. Absorption and fluorescence emission pattern of different molecular weight (MW) of HA conjugated dyes in ultrapure H_2O and 50/50 DMSO/H_2O. HA_{20k}-NIRF conjugates were illustrated in **Figure 1**. The excitation wavelength for absorption ranges from 600 nm to 900 nm, the fluorescence emission was collected between 790 nm to 900 nm. The concentrations of contrast agents were consistent in altered solvents.

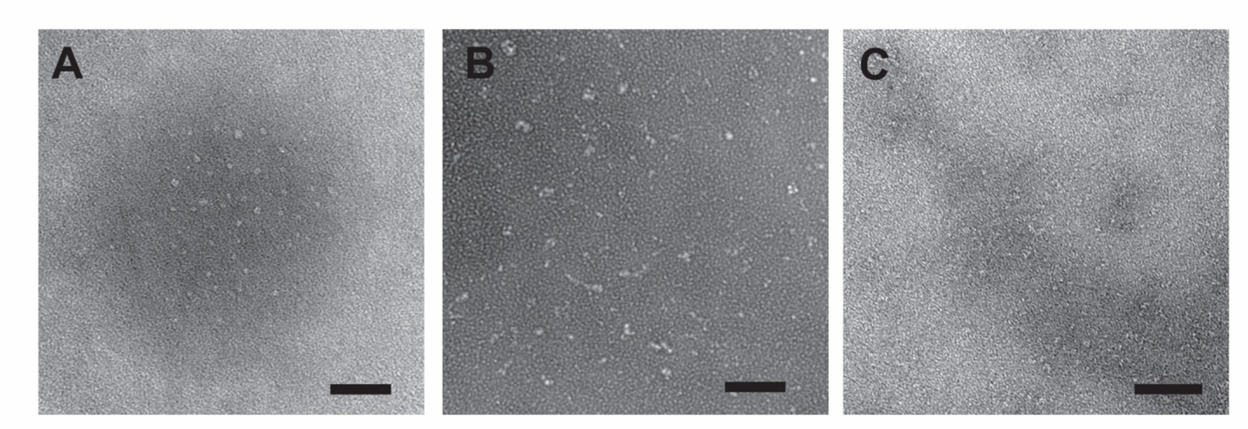

Figure **S2**. TEM images of BSA (A), BSA mixed with HA_{20k}-Cy7.5 (B), BSA mixed with HA_{20k}-IRDye800 (C) with negative staining, scale bar represents 100 nm.

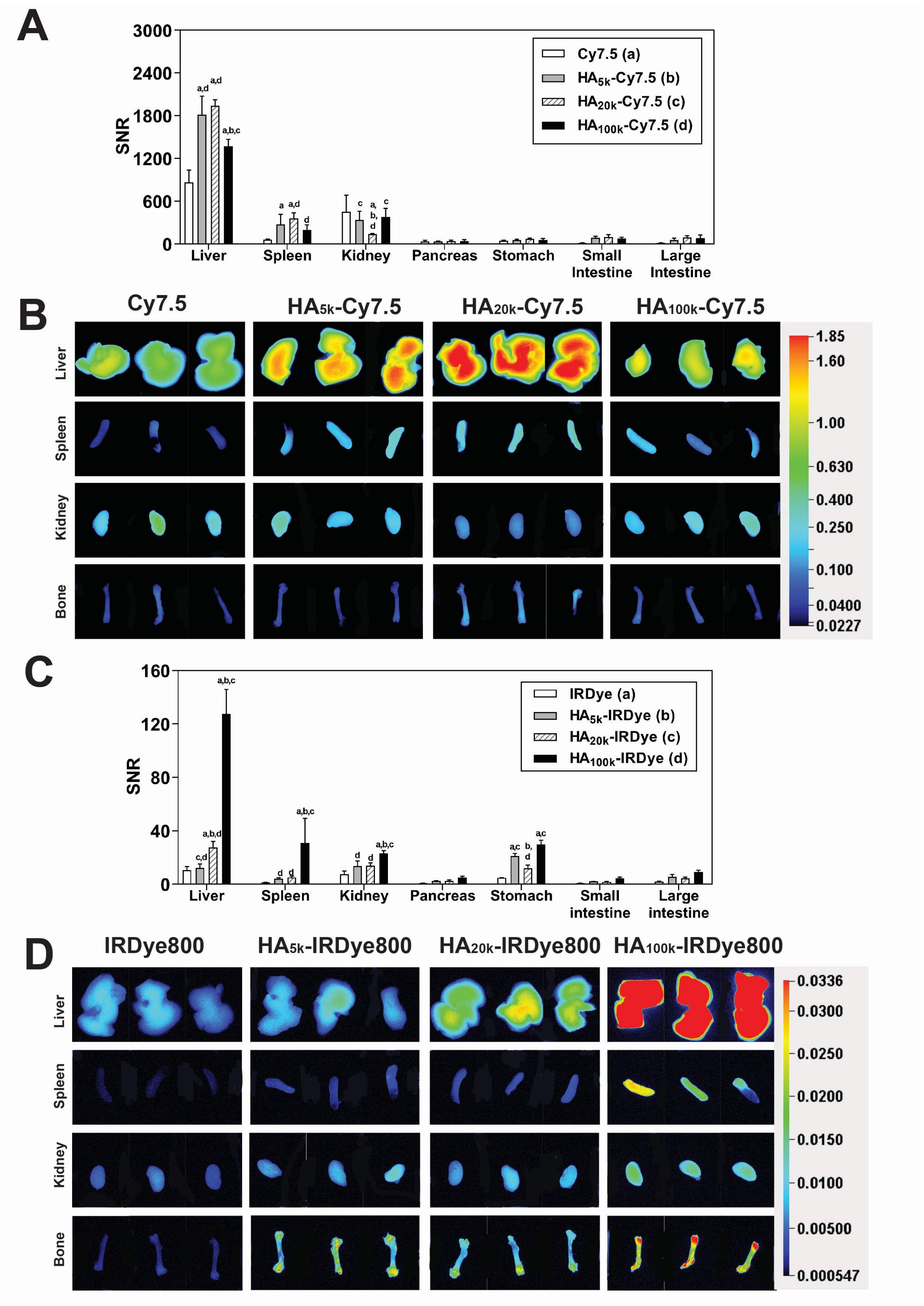
A
3000
2400
1800
1200
600
0
SNR
Cy7.5 (a)
HA5k-Cy7.5 (b)
HA20k-Cy7.5 (c)
HA100k-Cy7.5 (d)
Liver
Spleen
Kidney
Pancreas
Stomach
Small Intestine
Large Intestine
B
Cy7.5
HA5k-Cy7.5
HA20k-Cy7.5
HA100k-Cy7.5
Liver
Spleen
Kidney
Bone
1.85
1.60
1.00
0.630
0.400
0.250
0.100
0.0400
0.0227
C
160
120
80
40
0
SNR
IRDye (a)
HA5k-IRDye (b)
HA20k-IRDye (c)
HA100k-IRDye (d)
Liver
Spleen
Kidney
Pancreas
Stomach
Small intestine
Large intestine
D
IRDye800
HA5k-IRDye800
HA20k-IRDye800
HA100k-IRDye800
Liver
Spleen
Kidney
Bone
0.0336
0.0300
0.0250
0.0200
0.0150
0.0100
0.00500
0.000547

Figure S3. Relative biodistribution of HA-Cy7.5 and HA-IRDye800 in WT C57BL/6 mice 24 h post intravenous injection. (**A, C**) Quantitative organ biodistribution of HA-Cy7.5 and HA-IRDye800 were determined by the relative fluorescence of each organ from NIRF images, respectively. (**B, D**) Representative images of each HA MW_N and dye in organs of interest in WT C57BL/6 mice, which demonstrate the predominant clearance route of each contrast agent. Images of necropsied organs were acquired with the 800 nm channel of pearl trilogy small animal imaging system, interfering signal from neighboring organs was manually removed. N = 3, $^{a}p < 0.05$ as compared to free dye, $^{b}p < 0.05$ as compared to HA_{5k}-dye, $^{c}p < 0.05$ as compared to HA_{20k}-dye, $^{d}p < 0.05$ as compared to HA_{100k}-dye.

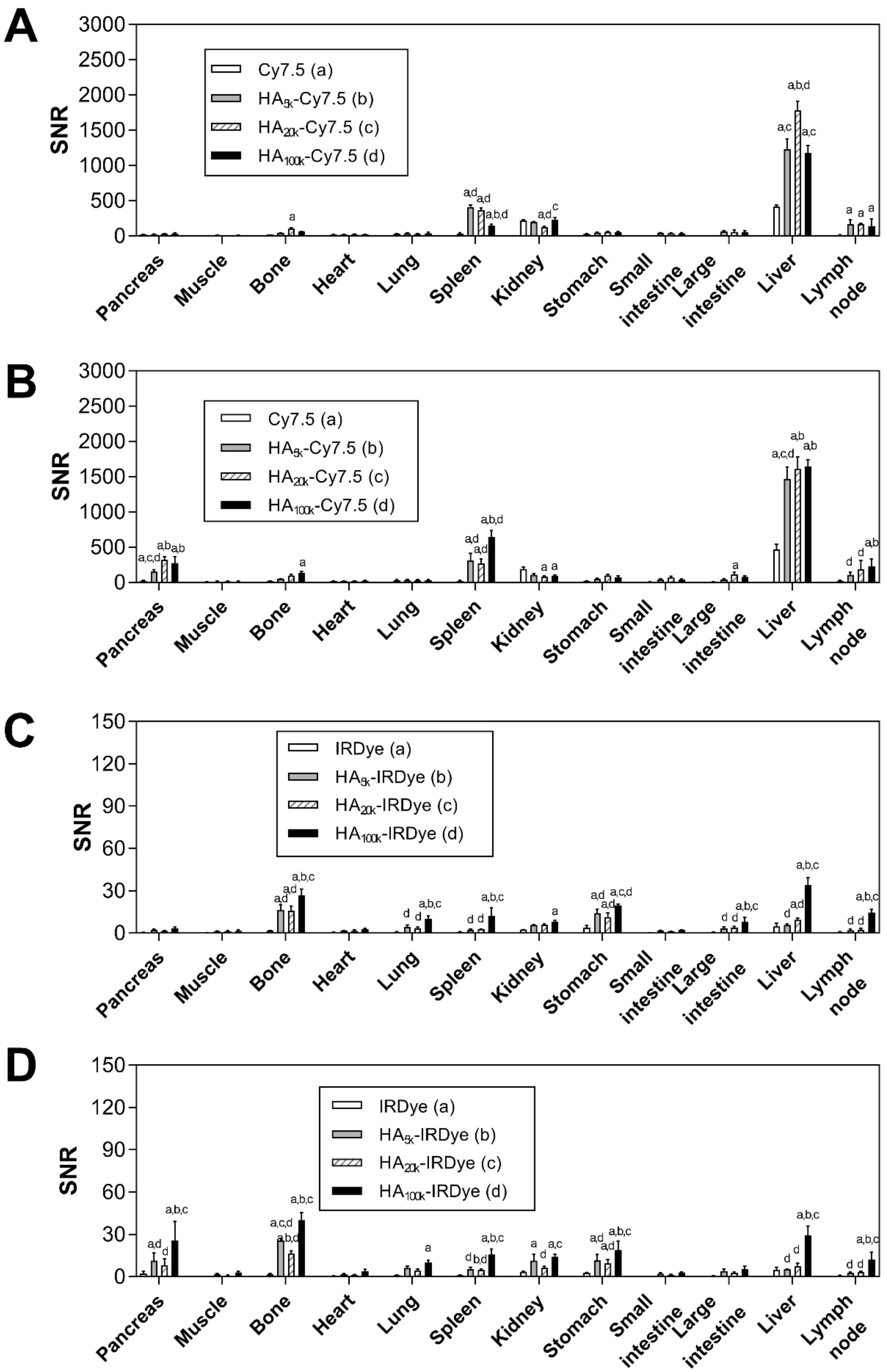

A
B
C
D
SNR
Cy7.5 (a)
HA5k-Cy7.5 (b)
HA20k-Cy7.5 (c)
HA100k-Cy7.5 (d)
IRDye (a)
HA5k-IRDye (b)
HA20k-IRDye (c)
HA100k-IRDye (d)
Pancreas
Muscle
Bone
Heart
Lung
Spleen
Kidney
Stomach
Small intestine
Large intestine
Liver
Lymph node

Figure S4. The biodistribution of HA-dye conjugates in WT C57BL/6 and pancreatic ductal adenocarcinoma (PDAC)-bearing mice. (A) Organ biodistribution of HA-Cy7.5 in wide type (WT) C57BL/6 mice 96 h post injection; (B) Organ biodistribution of HA-Cy7.5 in PDAC-bearing mice 96 h post injection; (C) Organ biodistribution of HA-IRDye800 in WT C57BL/6 mice 96 h post injection; (D) Organ biodistribution of HA-IRDye800 in PDAC-bearing mice 96 h post injection; signal to noise ratio (SNR) was calculated based of fluorescence of organ divided by the background noise, N = 5. $^{a}p < 0.05$ as compared to free dye, $^{b}p < 0.05$ as compared to HA_{5k}-dye, $^{c}p < 0.05$ as compared to HA_{20k}-dye, $^{d}p < 0.05$ as compared to HA_{100k}-dye.

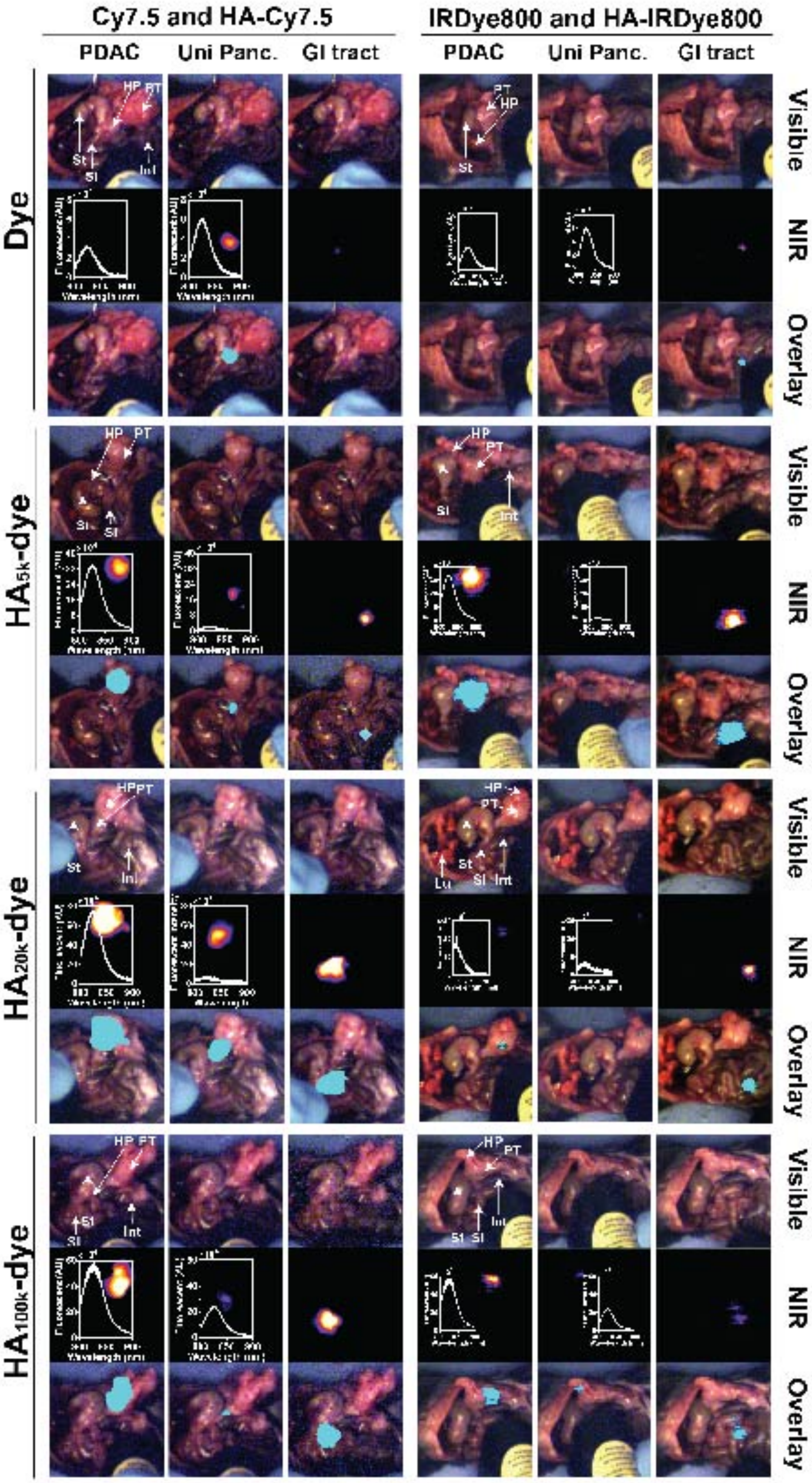

Cy7.5 and HA-Cy7.5
IRDye800 and HA-IRDye800
PDAC
Uni Panc.
GI tract
Dye
HA5k-dye
HA20k-dye
HA100k-dye
Visible
NIR
Overlay
HP
PT
St
SI
Int
Lu
Wavelength (nm)

Figure S5. Images from surgical navigation of pancreatic tumor contrast-enhanced with HA-dye 96 h post *i.v.* injection. Livers and spleens were removed due to high background signal from clearance, spleens that completely involved with PDAC were kept. PT = pancreatic tumor, HP = healthy (uninvolved) pancreas, St = stomach, SI = small intestine, Int = large intestine; each group was arranged into three rows: (*Top*) Color images show orthotopic PDAC (PT) and the location of the handheld spectroscopic pen, which provides NIR spectral information and serves as an excitation source for a widefield imaging system. (*Middle*) Pseudo-colored signal (fire) in the NIR channel of the widefield imaging system and the spectroscopic signal from within the contrast-enhanced region; (*Bottom*) NIR signal overlaid (cyan) onto the color image shows robust enhancement of syngeneic, orthotopic pancreatic cancer. Contrast enhancement can be observed with HA_{5k}-, HA_{20k}- and HA_{100k}-Cy7.5, HA_{5k}- and HA_{100k}-IRDye800 (see inset middle row).

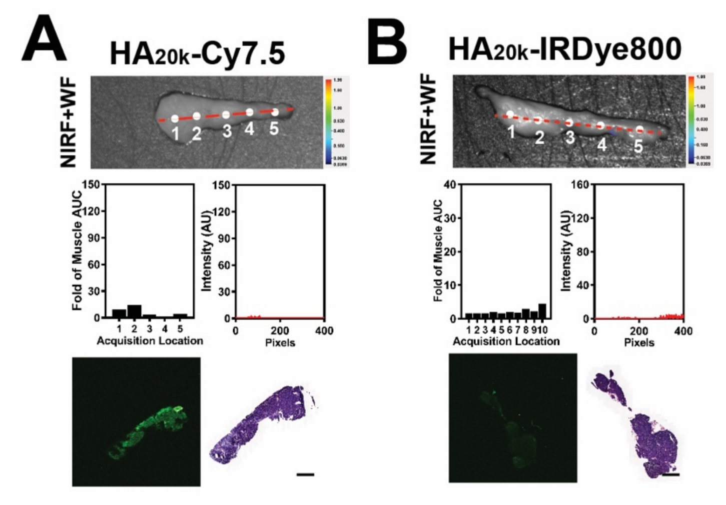

Figure S6. *Ex vivo* analysis of HA_{20k}-dye accumulation in healthy pancreas from W.T. C57BL/6 mice 24 h post injection. (*Top row*) NIRF channel and white field channel merged image of a representative pancreas marked with acquisition locations of the spectroscopic semi-quantification shown at the in *middle row*, fluorescence intensity excited with medium laser power and 1 s integration by the spectroscopic pen component of FIGS system; (*Bottom row*) plots of intensity

values along the red dashed line from the NIRF images quantified by ImageJ;

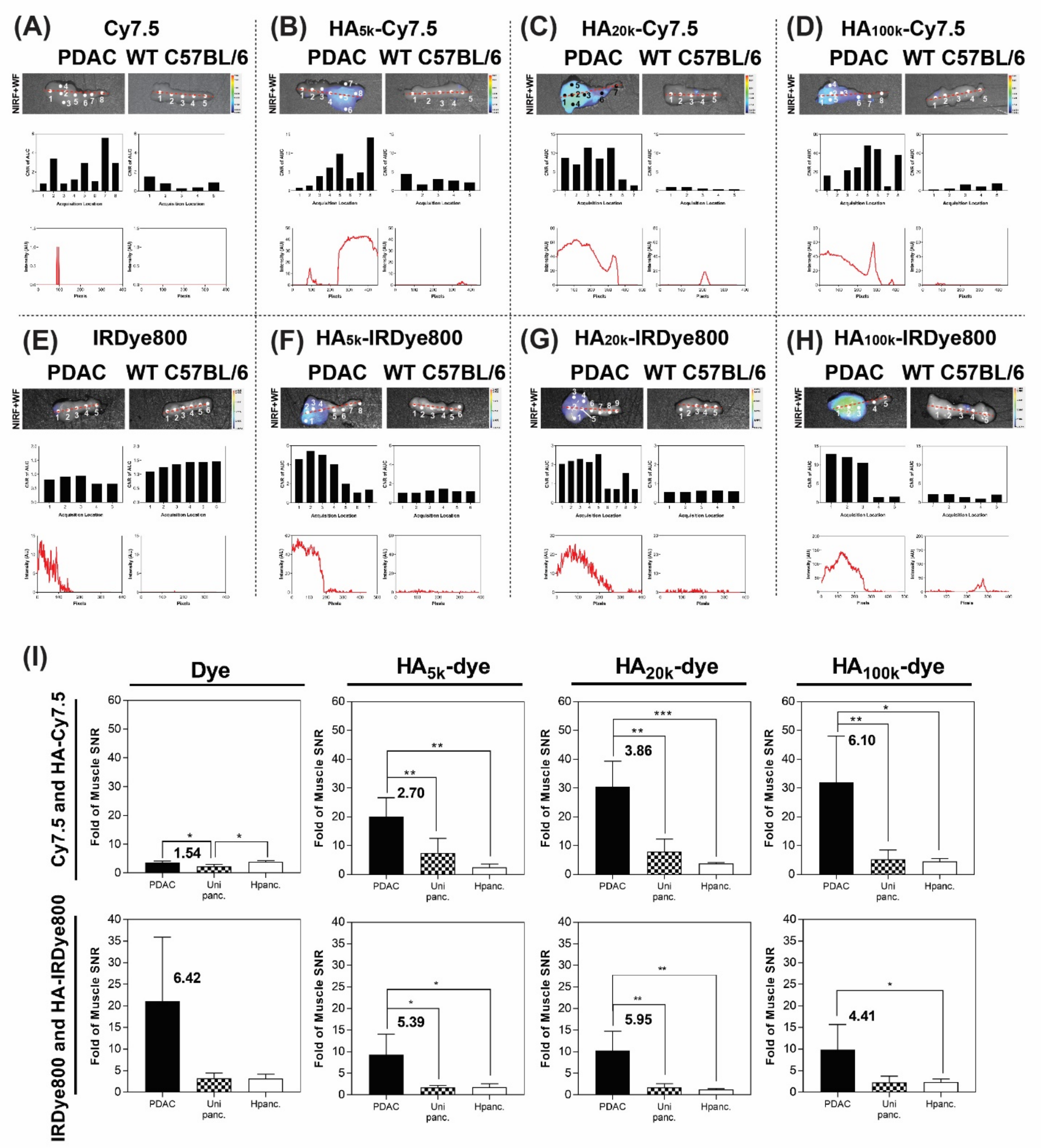

Figure S7. *Ex vivo* analysis of HA-dye accumulation in pancreatic tumor (*left column* for each group) or healthy pancreas from W.T. C57BL/6 mice (*right column* for each group) 96 h post

injection. (*Top row*) NIRF channel and white field channel merged image of a representative pancreas marked with acquisition locations of the spectroscopic semi-quantification shown at the in *middle row*, fluorescence intensity excited with medium laser power and 1 s integration by the spectroscopic pen component of FIGS system; (*Bottom row*) plots of intensity values along the red dashed line from the NIRF images quantified by ImageJ; (**I**) contrast within PDAC-bearing pancreas as determined by muscle SNR weighted SNR of PDAC, uninvolved pancreas (Uni panc.). and healthy pancreas (Hpanc) from WT C57BL/6. Data were collected 4 day post *i.v.* injection of contrast agents by tail vein. SNR value were calculated based of fluorescence intensity in NIRF images. ***$p < 0.001$, **$p < 0.01$, *$p < 0.05$. N = 4-5 for each group.

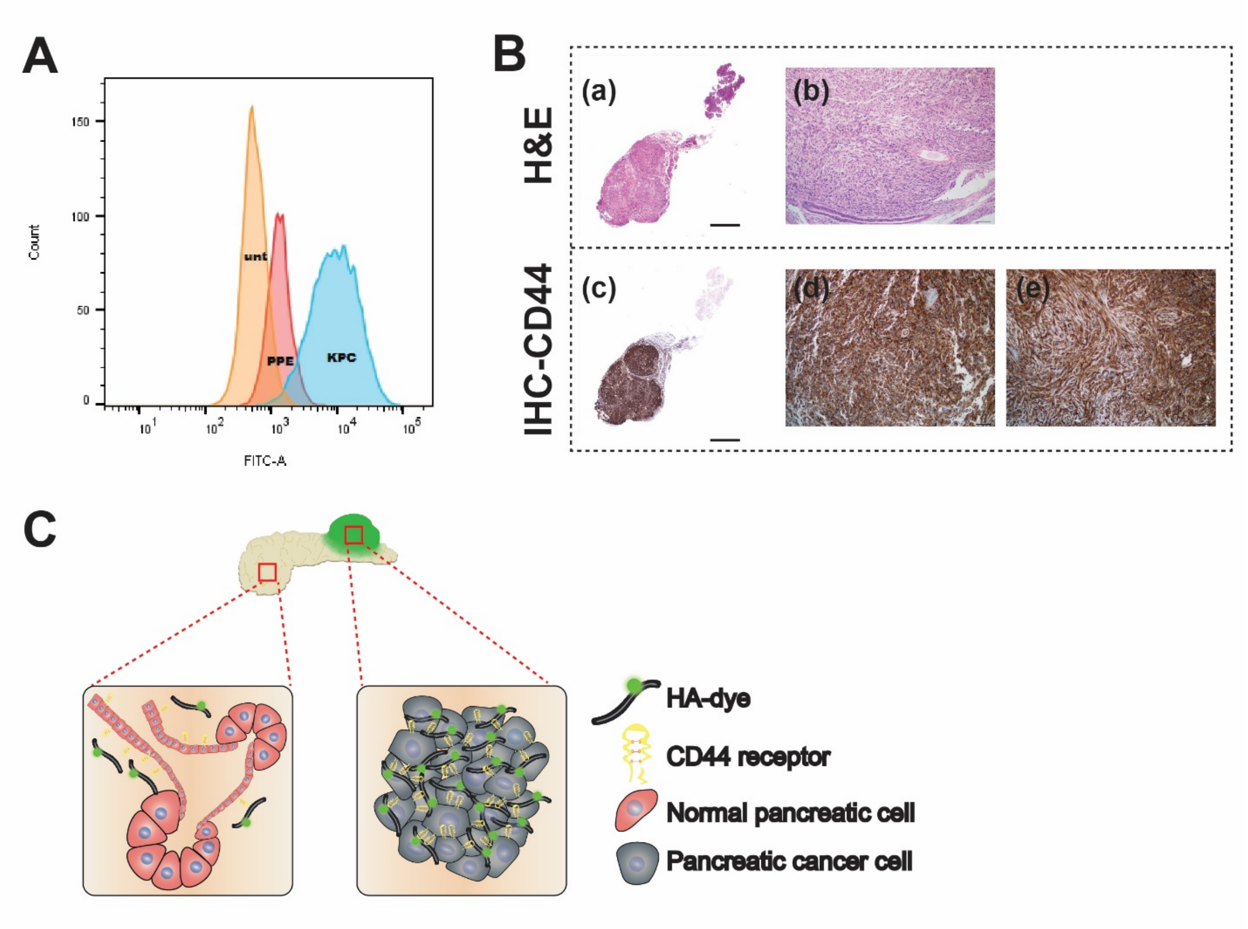

Figure S8. CD44 expression in PDAC cells *ex vivo* and *in vitro*. (A) Representative flow cytometry analytical histogram of untreated, primary pancreatic epithelial (PPE) and KPC cells single stained with CD44-FITC; (B) histology of PDAC-bearing murine pancreas; (a) scanned H&E stained murine PDAC; (b) mouse PDAC with 100 × magnification; (c) scanned CD44 stained slides of murine PDAC; (d) microscopic capture of mouse PDAC with 100 × magnification; (e) microscopic capture of mouse sarcomatoid with 100 × magnification. Scale bar represents 2 mm for scanned images and 10 μm in captured microscopic capture; (C) schematic representation of HA-dye bind to CD44 receptors, which were expressed intensively on pancreatic cancer cell but moderately on normal pancreatic cells.

Acute toxicity of HA_{20k}-NIRF was examined to further confirm their translational potential (**Figure S9-10**). Upon the administration of HA_{20k}-NIRF conjugates at 20 times of the dose used for imaging, a comprehensive diagnostic profile was determined for indications of toxicities in liver, kidney, bone, GI, pancreas. Disorders including malnutrition, dehydration, hyperglycemia/hypoglycemia were assessed. A slight increase of BUN for HA_{20k}-IRDye800 could be attributed to its renal clearance (**Figure S9**). Whereas reduced amount of total protein from HA_{20k}-Cy7.5 might be correlated with the strong protein adsorption (**Figure S9**). However, the lungs, kidneys, spleen, and heart were histologically unremarkable by H&E staining (**Figure S10**). Liver sections from each animal demonstrated mild, diffuse microvesicular steatosis within hepatocytes in the absence of lobular/portal inflammation, ballooning degeneration, or apoptosis. Brain sections overall were without abnormality with occasional animals showing scattered red neurons in Purkinje cells within the cerebellum.

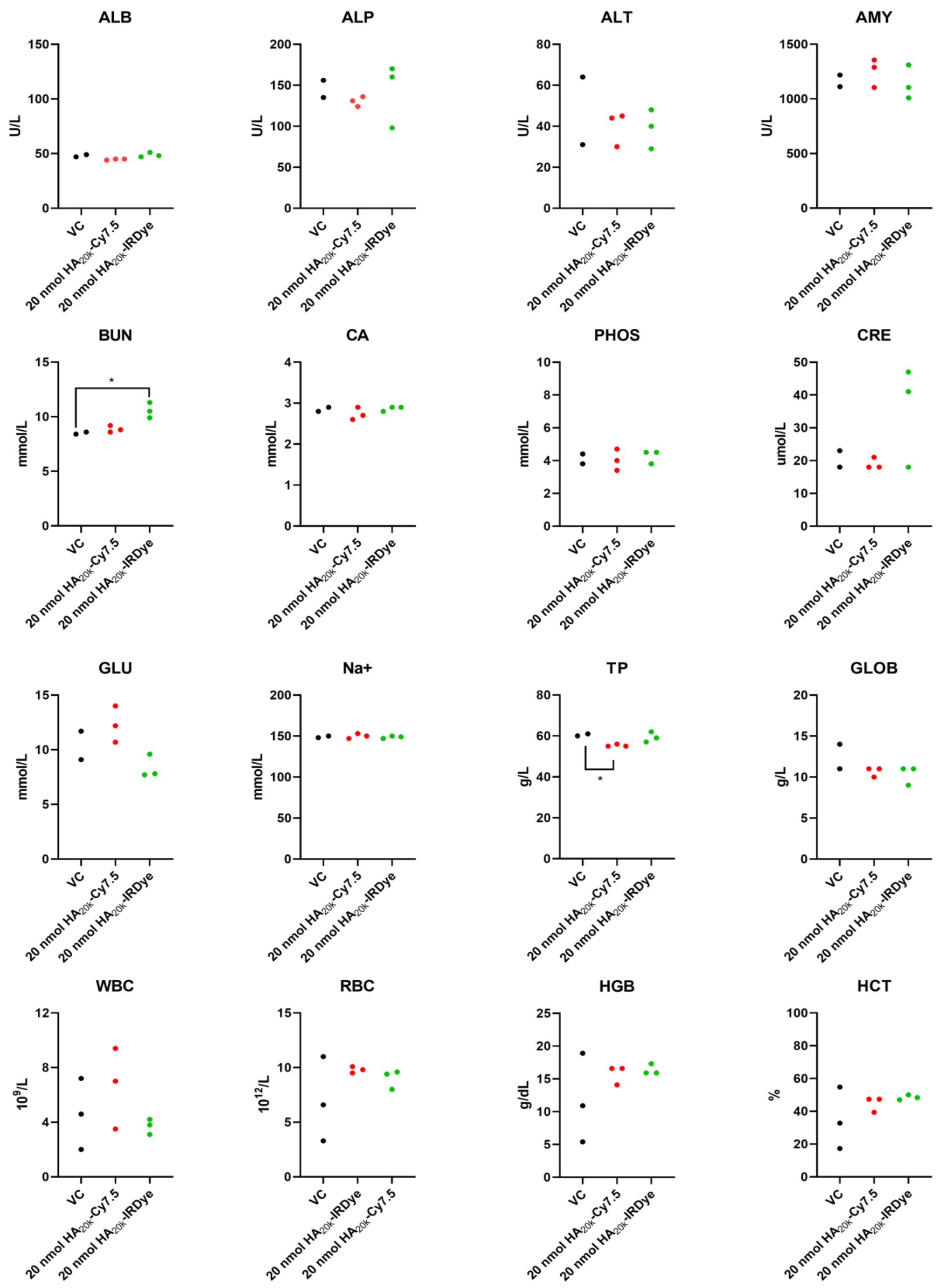

Figure S9. Comprehensive biochemical and hematological assessments for WT C57BL/6 mice injected with HA_{20k}-NIRF (equivalent to 20 nmol of free dye/mouse) or vehicle control for the acute toxicology study. *N* = 3. ALB, albumin; ALP, alkaline phosphatase; ALT, alanine aminotransferase; AMY, amylase; BUN, blood urea nitrogen; CA, calcium; PHOS, phosphorus;

CRE, Creatinine; GLU, glucose; TP, total protein; GLOB, globulin; WBC, white blood cells; RBC, red blood cells; HGB, hemoglobin; HCT, hematocrit.

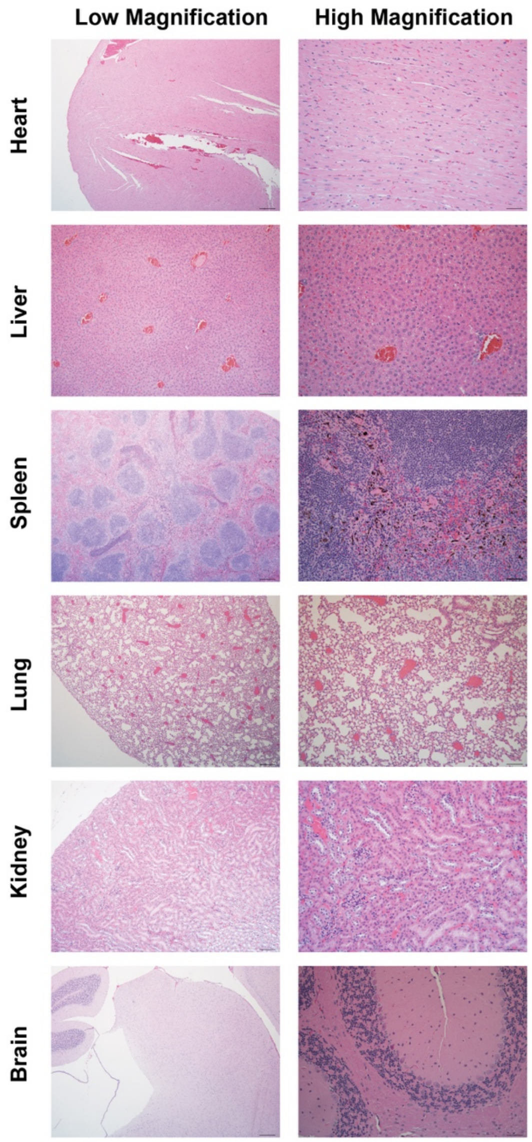

Figure S10. Representative images for the histological assessments of vital organs harvested from WT C57BL/6 mice injected with HA_{20k}-NIRF (equivalent to 20 nmol of free dye/mouse) or vehicle control. $N = 3$.

Table S1. Chemical Characteristics of HA-dye conjugates

	HA_{5k}-Cy7.5	HA_{20k}-Cy7.5	HA_{100k}-Cy7.5	HA_{5k}-IRDye800	HA_{20k}-IRDye800	HA_{100k}-IRDye800
Weight ratio (g/g) dye/polymer	0.57	0.15	0.10	0.26	0.05	0.02
Dyes per chain	1.23	1.15	0.56	1.18	0.69	1.40
Conjugating efficiency (%)	77.1	88.8	78.9	57.8	27.8	18.4
Yield (%)	37.3	33.3	21.8	45.6	68.5	75.5

Table S2. Ratio of HA-Cy7.5-treated pancreas or PDAC fluorescence divided by signal of surrounding organs 24 h post injection ($N = 5$)

	**W.T. C57BL/6**				**PDAC**			
	Cy7.5	HA_{5k}-Cy7.5	HA_{20k}-Cy7.5	HA_{100k}-Cy7.5	Cy7.5	HA_{5k}-Cy7.5	HA_{20k}-Cy7.5	HA_{100k}-Cy7.5
**Muscle**	2.31±0.50	4.64±1.29	4.46±1.21	4.44±1.27	3.59±1.68	27.56±4.83	33.88±11.98	40.76±20.90
**Stomach**	0.78±0.31	0.58±0.08	0.60±0.36	0.79±0.12	0.36±0.11	4.98±1.78	5.45±1.28	6.94±2.10
**Int**	2.38±0.41	0.42±0.20	0.41±0.07	0.62±0.39	1.77±0.75	3.67±1.19	5.77±1.50	10.06±3.09
**Liver**	0.04±0.01	0.02±0.003	0.02±0.01	0.03±0.01	0.05±0.02	0.25±0.06	0.51±0.11	0.43±0.25
**Kidney**	0.08±0.01	0.11±0.02	0.29±0.12	0.12±0.04	0.12±0.04	2.02±0.42	4.68±0.28	6.93±2.74
**Spleen**	0.56±0.17	0.14±0.03	0.12±0.08	0.24±0.12	0.69±0.40	1.18±0.41	1.76±0.32	1.11±0.40

Table S3. Ratio of HA-IRDye800-treated pancreas or PDAC fluorescence divided by signal of surrounding organs 24 h post injection ($N = 5$)

	W.T. C57BL/6				PDAC			
	IRDye	HA_{5k}-IRDye	HA_{20k}-IRDye	HA_{100k}-IRDye	IRDye	HA_{5k}-IRDye	HA_{20k}-IRDye	HA_{100k}-IRDye
**Muscle**	3.38±0.85	0.92±0.78	2.26±0.38	1.74±0.59	8.28±5.55	9.96±5.63	29.81±11.26	12.42±9.00
**Stomach**	0.17±0.03	0.39±0.58	0.23±0.08	0.17±0.05	1.06±0.59	1.54±0.97	3.50±1.32	1.04±0.77
**Int**	1.47±0.70	0.54±0.50	1.44±0.20	1.14±0.34	6.00±3.17	11.03±5.90	25.56±10.74	8.59±7.16
**Liver**	0.08±0.03	0.11±0.09	0.09±0.01	0.04±0.01	0.36±0.14	1.65±0.66	1.81±0.69	0.30±0.20
**Kidney**	0.12±0.02	0.10±0.08	0.19±0.03	0.22±0.06	0.52±0.22	1.14±0.48	1.96±0.34	1.06±0.65
**Spleen**	0.59±0.10	0.43±0.26	0.51±0.06	0.20±0.10	2.08±1.14	5.01±1.73	3.60±0.72	0.91±0.58

Abbreviations used are: Int: small intestines
